# Quality versus accuracy: result of a reanalysis of protein-binding microarrays from the DREAM5 challenge by using BayesPI2 including dinucleotide interdependence

**DOI:** 10.1186/1471-2105-15-289

**Published:** 2014-08-27

**Authors:** Junbai Wang

**Affiliations:** Pathology Department, Oslo University Hospital – Norwegian Radium Hospital, Montebello, Oslo, 0310 Norway

## Abstract

**Background:**

Computational modeling transcription factor (TF) sequence specificity is an important research topic in regulatory genomics. A systematic comparison of 26 algorithms to learn TF-DNA binding specificity in *in vitro* protein-binding microarray (PBM) data was published recently, but the quality of those examined PBMs was not evaluated completely.

**Results:**

Here, new quality-control parameters such as principal component analysis (PCA) ellipse is proposed to assess the data quality for either single or paired PBMs. Additionally, a biophysical model of TF-DNA interactions including adjacent dinucleotide interdependence was implemented in a new program - BayesPI2, where sparse Bayesian learning and relevance vector machine are used to predict unknown model parameters. Then, 66 mouse TFs from the DREAM5 challenge were classified into two groups (i.e. good vs. bad) based on the paired PBM quality-control parameters. Subsequently, computational methods to model TF sequence specificity were evaluated between the two groups.

**Conclusion:**

Results indicate that both the algorithm performance and the predicted TF-binding energy-level of a motif are significantly influenced by PBM data quality, where poor PBM data quality is linked to specific protein domains (e.g. C_2_H_2_ DNA-binding domain). Especially, the new dinucleotide energy-dependent model (BayesPI2) offers great improvement in testing prediction accuracy over the simple energy-independent model, for at least 21% of analyzed the TFs.

**Electronic supplementary material:**

The online version of this article (doi:10.1186/1471-2105-15-289) contains supplementary material, which is available to authorized users.

## Background

Recently, a comprehensive evaluation of 26 algorithms, for modeling transcription factor (TF) sequence specificity in *in vitro* protein-binding microarray (PBM) data [1], was published by DREAM5 (the Dialogue for Reverse Engineering Assessments and Methods) consortium. Many interesting results were revealed through this work. For example, mononucleotide position weight matrices (PWM) methods perform similarly to more advanced dinucleotide PWM algorithms for modeling TF sequence specificity, and inferred binding energy-level of a motif has little effect on overall prediction accuracy. This study also briefly mentioned that PBM data quality may have a strong influence on algorithm performance across 66 mouse TFs. However, the actual data quality of the examined PBMs in the DREAM5 challenge (i.e. 66 training PBMs and 66 testing PBMs for the mouse TFs) is not investigated systematically. Generally, the microarray experiment is known for containing many kinds of biases [2, 3] such as nonlinearity, saturation, and dynamic range problems for the signal intensity. In DREAM5 challenge, for a pair of training and testing PBM experiments, two different array designs were used for a mouse TF. However, 8-mers that were used to compute the 8-mer median intensities for every PBM are identical. This unique feature provides an opportunity to assess the PBM data quality [4]. For instance, if both training and testing PBM experiments in good data quality, then the observed 8-mer median intensities between the training and testing PBMs will have good agreement. On the contrary, if one of the PBMs yields poor data quality, then the 8-mer median intensities between two PBMs will not match well. Consequently, the testing prediction accuracy is not a true reflection of the algorithm performance if paired PBMs have poor measurement agreements. In other words, computational algorithms will not predict a binding signal that only exists in the testing PBM experiment but it does not appear in the training PBM data, and vice versa. Thus, it is important to develop PBM quality-control parameters that can evaluate the data quality for either single or paired PBMs.

Free-energy-based biophysical modeling TF sequence specificity, from detailed theoretical studies [5–7] to rapid computational development in real applications [8–11], have been investigated for many years and several computer programs are publically available now [11–14]. Recently, dependent energy correction such as dinucleotide interdependence was also incorporated into TF-binding energy by BEEML-PBM and FeatureREDUCE [1]. In the DREAM5 challenge, performance of the dinucleotide-dependent model of the two new programs is not improved greatly over the simple energy-independent model (i.e. <10% of examined TFs were benefited by the energy-dependent model; increase in correlation coefficient > 0.05 [1]). However, in many earlier studies, sequence dependencies in TF-binding sites were widely observed [15–18]. Particularly, energy-dependent model needs to fit a large number of unknown model parameters, which often encounters the over-fitting data problem that impairs the algorithm performance [19]. Additionally, if the input data is large, then there is a memory issue to R and MATLAB programs which suffer from extremely slow computation (i.e. BEEML-PBM and many other programs in the DREAM5 challenge [1]). Therefore, it is worthy to design a novel algorithm which implements the dependent energy correction in an efficient programming language. Then, PBMs of 66 mouse TFs from the DREAM5 challenge can be reanalyzed by the new program. It may help revealing whether the limitation of previous algorithms hampers the discovery of motifs that contain nucleotide dependency in the binding sites.

Motivated by the above-mentioned challenges, new quality-control parameters for both single and paired PBMs, and a novel C implementation of biophysical modeling protein-DNA interactions including dinucleotide interdependence (BayesPI2) are presented here. The new methods and programs were applied on 66 mouse TFs in *in vitro* PBM experiments from the DREAM5 challenge. Overall, four major questions are investigated in this work: 1) the true data quality of paired PBM experiments for 66 mouse TFs; 2) the association between the PBM data quality and the algorithm performance; 3) whether the binding energy-dependent model offers a great improvement over the simple energy-independent model in testing prediction accuracy; 4) whether the low binding energy-level of a motif is a real biological phenomenon or a bias due to the data quality and the algorithm limitation.

## Results

### PBM data quality of two mouse TFs

The new PBM quality-control parameters were first tested at TF_7 (Mix) and TF_63 (Zkscan5), since they are extreme cases in the previous algorithm performance comparison [1]. For example, in Figure two of the original publication, 66 mouse TFs were sorted in decreasing order by the mean final algorithm performance scores, TF_7 and TF_63 were ranked as the first and the last TF, respectively. It suggests that the majority of evaluated algorithms performed significantly better at TF_7 than at TF_63 in the original study. In other words, the PBM data quality of TF_7 may be much better than that of TF_63. Thus, a quality analysis of the above-mentioned two TFs may tell the usefulness of new PBM quality-control parameters.

**Figure 1:**
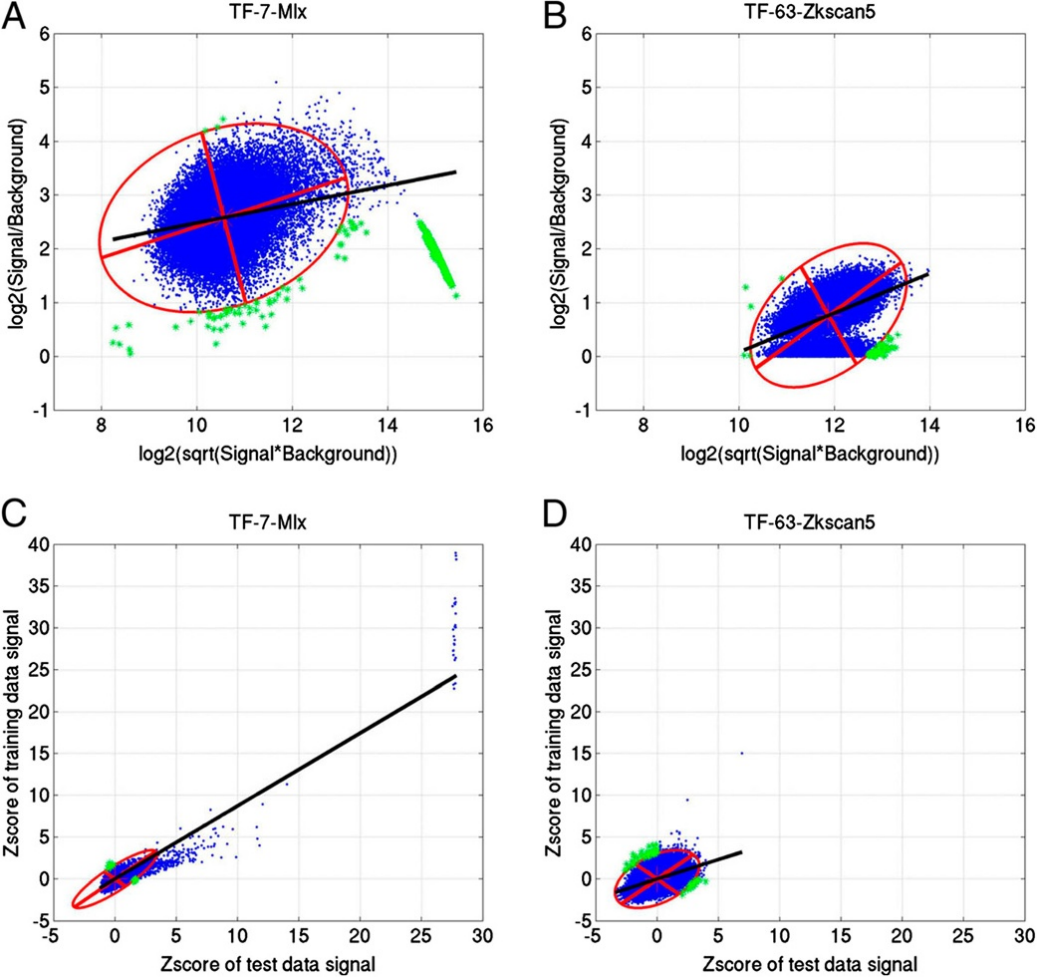
**PBM data quality for TF_7 (Mix) and TF_63 (Zkscan5). A** and **B** are MA scatter plots of TF_7 and TF_63, respectively. **C** and **D** are scatter plots of Z-score transformed 8-mer median intensities between a pair of training and testing PBM experiments for TF_7 and TF_63, respectively. In the figure, the red ellipses are 99.73% limit of PCA quality-control ellipses, the two red smooth lines are the length of the major and minor axes of the PCA ellipses, the black smooth lines are fitted linear regression lines, and the green data points are observations that may be out of the quality control (i.e. data point outside of the PCA ellipse and one of the observations is below sample mean).

Figures 1A and 1B show the MA plots [2] of single PBM quality (training experiment) for TF_7 (Mix) and TF_63 (Zkscan5), respectively. In the MA plots, the higher the algorithm performance rank order of a TF, the longer the length of the major axis of the PCA ellipse (i.e. ~5.3 and ~3.6 for TF_7 and TF_63, respectively). The major axis of the PCA ellipse is related to the orthogonal regression line between the background signal and PBM binding signal, which indicates the dynamical range of measured PBM signal intensities. If a PBM experiment has larger dynamical range, then better separation between the noise background signal and the true binding signal is achieved. Consequently, a higher TF rank order will be obtained in algorithm performance evaluation. The present results support assumption that the longer the length of the major axis the better the PBM data quality.

Figure 1C and 1D illustrate the quality of paired PBMs for TF_7 and TF_63, by applying PCA ellipse on the scatter plot of Z-score transformed and log normalized 8-mer median intensities between the two PBMs. In the scatter plots, the lengths of both the major and minor axes of the PCA ellipse are quite different between TF_7 (i.e. 9.4 and 2.4; Figure 1C) and TF_63 (i.e. 8.3 and 5.0; Figure 1D). This is consistent with the previous hypothesis in single PBM experiment that the lengths of the major and minor axes reflect the dynamical range of PBM signal intensities, and the difference of 8-mer median intensities between paired PBMs, respectively. Put differently, if a paired PBMs has good data quality (i.e. TF_7), then a PCA ellipse with long major axis but short minor axis will be expected. For paired PBMs, correlation coefficients of normalized 8-mer median intensities are also quite different between the good-quality PBMs (i.e. 0.87; TF_7) and the bad-quality ones (i.e. 0.46; TF_63). Nevertheless, it is not an indicator of measurement agreement between the two PBMs, because correlation coefficients measure the strength of a relationship between the two variables, and data with obvious poor agreement can produce high correlations [20]. Therefore, the new PBM quality-control parameters not only provided a visual inspection of data quality for either single or paired PBMs, but also suggested that TF rank order of algorithm performance comparison [1] is associated with the data quality of both training and testing PBMs.

### PBM data quality of 66 mouse TFs

**Figure 2:**
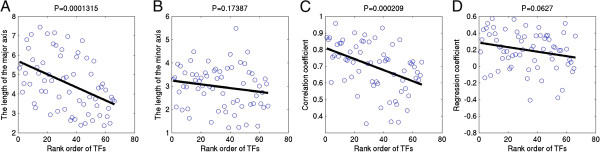
**Scatter plots of algorithm performance rank order versus PBM training data quality. A**, **B**, **C**, and **D** show scatter plots of algorithm performance rank order of 66 mouse TFs versus the length of the major axis of the PCA ellipse (i.e. 99.73% limit of PCA quality control ellipse), the length of the minor axis of the PCA ellipse, correlation coefficient between signal intensities and background intensities, and regression coefficients, respectively. Both PCA ellipse and regression coefficient are based on MA scatter plots for PBM training datasets. In the figure, the black smooth lines are fitted linear regression lines to the scatter plots, and the P-values of the regression lines are indicated at the top of each figure. The rank order of TFs (i.e. 66 TFs were sorted in decreasing order by mean final algorithm performance scores) is adopted from Figure 2 of the DREAM5 challenge paper.

Encouraged by the above observations, it is necessary to investigate all 66 TFs that have paired PBMs. First, scatter plots of TF rank order vs. the single PBM (training data) data quality are shown in Figure 2, where x-axis is the sorted rank order of 66 TFs based on the algorithm performance comparison in Figure 2 of original paper [1], and y-axis is the single PBM quality parameter for 66 training PBM experiments such as the length of the major and minor axes of the PCA ellipse (Figure 2A and 2B), correlation coefficient (Figure 2C), and regression coefficient (Figure 2D). A linear regression line was fitted to every scatter plot, where P-values to regression coefficients for the length of the major axis of the PCA ellipse, the correlation coefficient, and the regression coefficient are P < 0.00014, P < 0.00021, and P < 0.063, respectively. The results suggest that the quality of training PBM experiments is significantly correlated to the TF rank order of mean algorithm performance comparison. Nevertheless, in a similar study by 66 testing PBM experiments, most of the single PBM quality parameters are not linked to the TF rank order Additional file 1: Figure S1, except for the correlation coefficient between the signal intensities and the background intensities (P < 0.0022). It indicates that the algorithms may have been learning some background signals, since in general they performed best on training or testing sets where the signal and background intensities are highly correlated. For that reason, algorithm performance comparison of the original paper [1] was swayed by the quality of PBM training data (i.e. Figure 2A and 2C).

**Figure 3:**
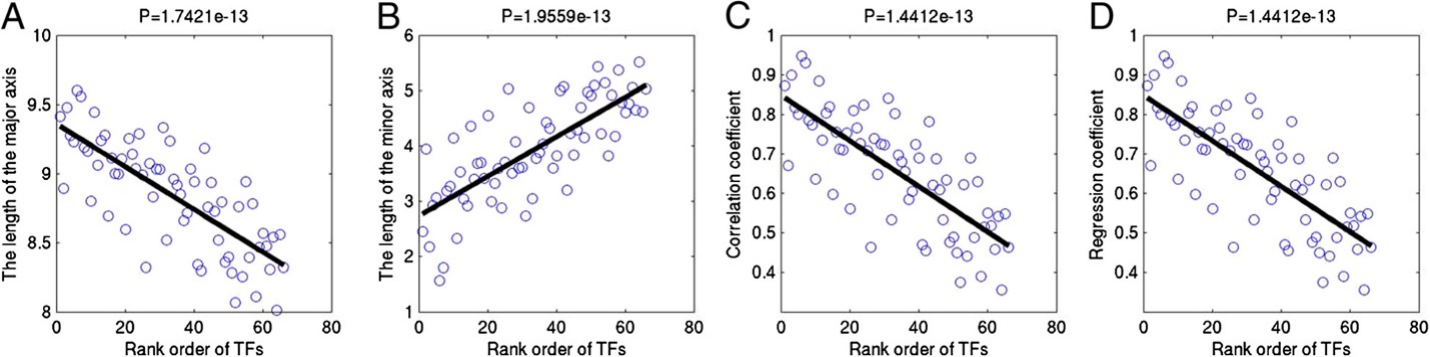
**Scatter plots of algorithm performance rank order versus agreement of paired PBMs. A**, **B**, **C**, and **D** show scatter plots of algorithm performance rank order versus the length of the major axis of the PCA ellipse (i.e. 99.73% limit of PCA quality-control ellipse), the length of the minor axis of the PCA ellipse, correlation coefficient of normalized 8-mer median intensities between a pair of training and testing PBM experiments, and regression coefficients, respectively. Both PCA ellipse and regression coefficient are based on scatter plots of Z-score transformed 8-mer median intensities between a pair of training and testing PBM experiments. In the figure, the black smooth lines are fitted linear regression lines to the scatter plots, and the P-values of the regression lines are indicated at the top of each figure. The rank order of TFs (i.e. 66 TFs were sorted in decreasing order by mean final algorithm performance scores) is adopted from Figure 2 of the DREAM5 challenge paper.

Then, scatter plots of TF rank order versus the paired PBM quality parameters are illustrated in Figure 3, where a linear regression line was fitted to each plot. P-values to the regression coefficients are P < 1.75 × 10^-13^, P < 1.96 × 10^-13^, P < 1.45 × 10^-13^, and P < 1.45 × 10^-13^ for the length of the major axis (Figure 3A), and minor axis (Figure 3B) of the PCA ellipse, the correlation coefficients (Figure 3C), and the regression coefficients (Figure 3D) of normalized 8-mer median intensities between training and testing PBMs, respectively. The results are very interesting because all quality-control parameters of paired PBMs are significantly correlated to the TF rank order according to the mean algorithm performance comparison. It appears that the deterioration of mean algorithm performance across the 66 TFs (i.e. Figure 2 of the original publication [1]) is largely due to the decrease in agreement between the training and the testing PBMs. Specifically, computational methods for modeling TF sequence specificity are extremely sensitive to the data quality of both training and testing PBM experiments.

### Classifying 66 mouse TFs into two groups based on PBM data quality

So far, the results of analyzing 66 mouse TFs imply that methods for modeling TF sequence specificity are strongly affected by the PBM data quality. It is better to group 66 TFs into two clusters (i.e. good versus bad quality) by PBM quality-control parameters, then to reevaluate the algorithm performance (i.e. BayesPI2 energy-independent model versus the energy-dependent model including dinucleotide dependence). As a consequence, 66 mouse TFs from the DREAM5 challenge were assigned to two clusters by applying unsupervised fuzzy neural gas methods on the single PBM quality parameters (i.e. training PBM experiment). Comparing to the known TF rank order from original work [1], the best classifications were achieved by two parameters (i.e. correlation coefficient between normalized signal intensities and background intensities, and the length of the major PCA axis). That is consistent with the earlier observations in Figure 2A and 2C. The clustering result is an average of ten times classifications based on the above-mentioned two-quality parameters, where cluster one contains more good-quality PBM experiments than that in cluster two. For example, ~79% of 24 TF that grouped in the first cluster belong to the top half of ranked TFs (i.e. TF rank order from 1 to 33), and ~67% of 42 TF that assigned to the second cluster are in the bottom half of ranked TFs (i.e. TF rank order from 34 to 66). In summary, the quality of training PBM experiments influences the algorithm performance comparison. In other words, the algorithm performance on testing data may be predicted by the corresponding training data quality.

Then, the same 66 mouse TFs were classified into two clusters based on paired PBM quality parameters (i.e. agreement between training and testing PBMs). A combination of different quality parameters (i.e. the length of the major and minor axes of the PCA ellipse, regression coefficient, and correlation coefficients of normalized 8-mer median intensities between training and testing PBMs) were tested, and the best classification was obtained by using the lengths of both the major and minor axes of PCA ellipse, which characterize the dynamical range of PBM signal intensities and the difference of 8-mer median intensities between the two PBMs, respectively. The average of ten times classifications of 66 TFs by the two parameters is shown in Table 1 and Additional file 1: Table S1 for good and bad PBMs, respectively. In the tables, TFs were evenly assigned to two clusters: 34 TF were grouped in cluster one (Table 1) where ~79% of them have algorithm performance rank order from 1 to 33; and 32 TF were grouped in cluster two (Additional file 1: Table S1) where ~81% of them have algorithm performance rank order between 34 and 66 [1]. The results imply that the TFs of cluster one (Table 1) were mostly measured by paired PBMs with good data quality, but the TFs of cluster two (Additional file 1: Table S1) were frequently observed under poor-quality PBM experiments. Particularly, the classification based on the agreement of paired PBMs is much better than that done by single PBM data quality. It demonstrates that the quality of both training and testing PBMs plays a pivotal role in evaluating algorithm performance for computational methods to model TF sequence specificity. Thus, the new classification based on the agreement of paired PBMs will be utilized in future data analysis.

**Table 1:** **Prediction results of TFs with good PBM quality by using BayesPI2 energy-independent model and energy-dependent model including dinucleotide interactions**

	TF family	Rank	CorrCoef (Ind)	Length (Ind)	Number (Ind)	CorrCoef (Dep)	Length (Dep)	Number (Dep)
TF_7	bHLH	1	0.78	13	1	0.786	10	1
TF_26	bHLH	2	0.66	10	1	0.67	13	1
TF_56	C2H2 Z F(4)	3	0.76	12	1	0.788	13	1
TF_55	AT hook	4	0.8	8	1	0.8	9	1
TF_17	NR	5	0.76	9	1	0.757	8	1
TF_11	NR	6	0.826	10	1	0.833	10	1
TF_16	Myb/SANT	7	0.808	10	1	0.817	9	1
TF_31	C2H2 ZF (13)	8	0.585	12	1	0.59	11	1
TF_15	Pou + Homeo	9	0.62	11	1	0.655	11	1
TF_45	Myb/SANT	11	0.8	12	1	0.78	11	1
TF_42*	**Forkhead**	12	**0.75**	12	1	**0.805**	13	1
TF_64	C2H2 ZF (3)	13	0.75	8	1	0.75	10	1
TF_52	NR	14	0.807	12	1	0.79	10	1
TF_3*	**Forkhead**	16	**0.67**	10	1	**0.724**	11	1
TF_27*	**bZIP**	17	**0.526**	9	1	**0.635**	11	1
TF_18	Sox	18	0.638	8	1	0.677	8	1
TF_22*	**T-box**	19	**0.675**	9	1	**0.746**	12	1
TF_47	Homeo	21	0.726	12	1	0.767	11	1
TF_44	GATA	22	0.633	12	1	0.68	10	1
TF_28	C2H2 ZF (8)	23	0.58	11	1	0.6	11	1
TF_13*	**Pou + Homeo**	24	**0.584**	9	1	**0.675**	13	1
TF_5	C2H2 ZF (3)	25	0.59	10	1	0.618	11	1
TF_43	Forkhead	27	0.577	10	1	0.614	12	1
TF_19	Sox	29	0.559	8	1	0.59	11	1
TF_39	C2H2 ZF (3)	30	0.63	11	1	0.668	13	1
TF_51*	**Pou + Homeo**	31	**0.615**	12	1	**0.67**	13	1
TF_23	T-box	33	0.64	12	1	0.66	13	1
TF_12*	**NR**	35	**0.55**	13	1	**0.606**	13	1
TF_49	NR	34	0.675	10	1	0.676	11	1
TF_53*	**RFX**	39	**0.696**	12	1	**0.77**	13	1
TF_14	Myb/SANT	40	0.6	8	1	0.62	9	1
TF_48	NR	43	0.755	12	1	0.78	12	1
TF_38	DM	45	0.67	9	1	0.689	8	1
TF_32	C2H2 ZF (6)	55	0.587	9	1	0.62	8	1

In the table, the 34 TFs were classified by applying fuzzy neuronal gas algorithm on the paired PBM quality-control parameters (i.e. the length of the major and minor axes of the PCA ellipses), where a good agreement between training and testing PBMs indicates good PBM data quality; Rank means TFs are sorted in decreasing order of their final performance score across all tested algorithms in Figure 2 of original publication [1]; CorrCoef , Length, and Number are Pearson correlation between predicted intensities and testing probe intensities, the length of motif, the first or second motif, respectively; (Ind) and (Dep) represent BayesPI2 energy-independent model and energy-dependent model including dinucleotide interaction, respectively; TFs marked by star and bold text indicate that the increase in Pearson correlation coefficient is greater than 0.05 by using BayesPI2 energy-dependent model including dinucleotide interaction energies.

### Verification of BayesPI2 energy-dependent model including dinucleotide interaction energies

**Figure 4:**
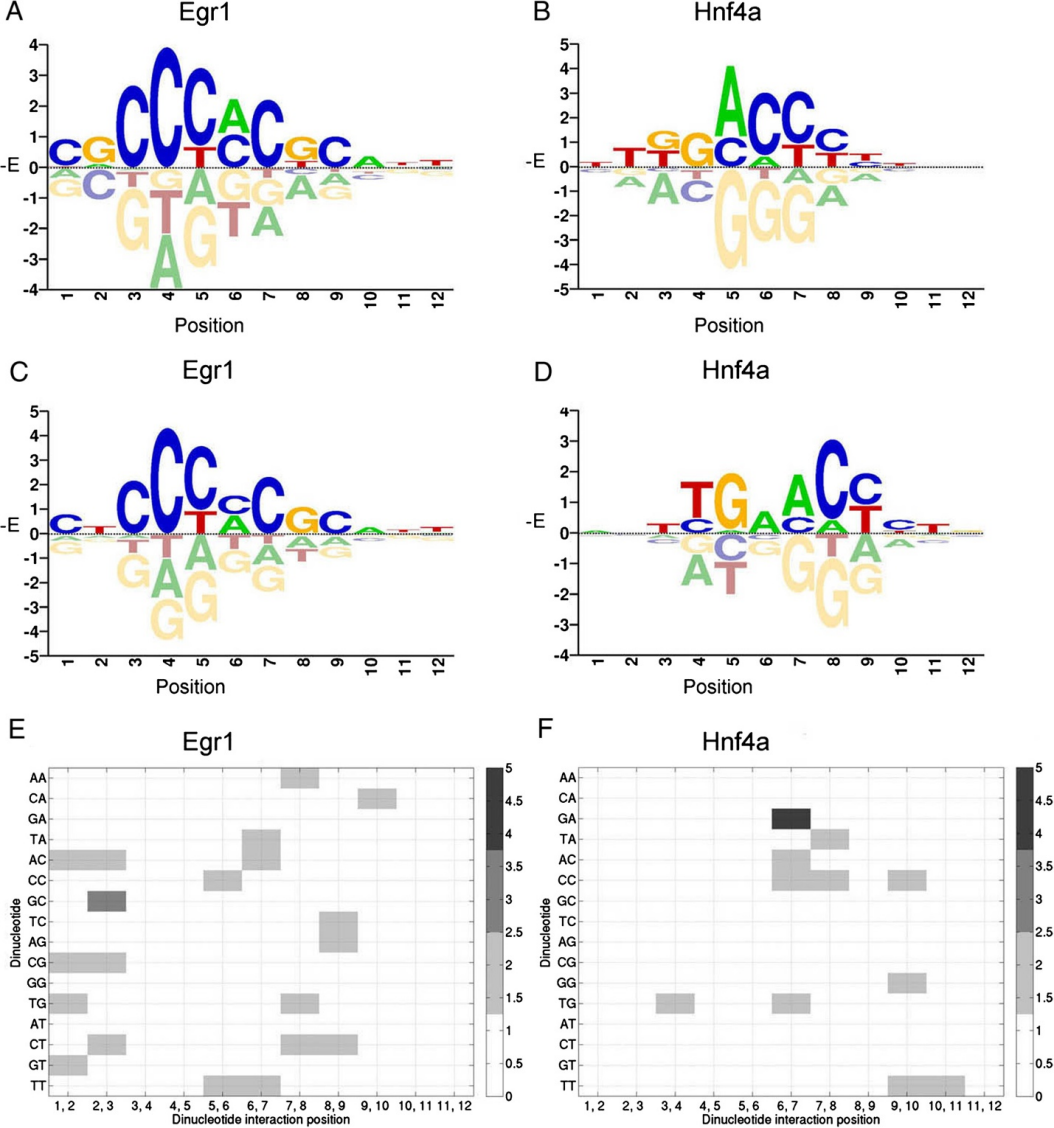
**Predicted binding energy matrices for Egr1 and Hnf4a. A** and **B** are predicted PBEMs for Egr1 and Hnf4a, respectively, by BayesPI2 energy-independent model. **C** and **D** are predicted PBMEs for Egr1 and Hnf4a, respectively, by BayesPI2 energy-dependent model including dinucleotide interdependence. Figure 4E and 4F are predicted energy-dependent matrices of dinucleotide interactions (i.e.  where  is the dinucleotide-dependent energy correction) for Egr1 and Hnf4a, respectively, by BayesPI2 energy-dependent model. In **E** and **F**, the dark and the light gray color represent the high and the low binding energy-level, respectively.

In this study, biophysical modeling of protein-DNA interaction with dinucleotide interdependency is implemented in C - BayesPI2, by using techniques similar to sparse Bayesian learning and relevance vector machine. To test the new program, it was evaluated by two PBM datasets (Egr1 and Hnf4a), which are known to contain nucleotide interdependent effects on the binding affinities of TFs [17, 21, 22]. First, the protein-binding energy matrices (PBEMs) of both Egr1 and Hnf4a were estimated, by applying BayesPI2 on Z-score transformed and log-normalized probe intensities of one of the replicate PBMs (i.e. motif length ranges from 7 to 12). Then, the predicted PBEMs were used to estimate the TF-binding intensities on the other replicate PBMs. Binding energy matrices predicted by the BayesPI2 energy-independent model, which result in the highest correlation coefficient between the predicted intensities and the testing probe intensities, are shown in Figure 4A and 4B for Egr1 (correlation coefficient 0.74) and Hnf4a (correlation coefficient 0.58), respectively. The best PBEMs calculated by BayesPI2 dinucleotide energy-dependent model are displayed in Figure 4C and 4D for Egr1 (correlation coefficient 0.77) and Hnf4a (correlation coefficient 0.71), respectively. The corresponding dinucleotide interaction energies are shown in the heatmaps, Figure 4E and 4F. It is clear that BayesPI2 energy-dependent model including dinucleotide interdependence improves testing prediction accuracy for both TFs. Especially, for Hnf4a, the improvement is striking (i.e. the difference of correlation coefficients between the energy-dependent and the energy-independent model is greater than 0.1), and the strongest dinucleotide interaction occurs at positions 6 and 7 (Figure 4D and 4F), which is consistent with a previous study [22] that applied BEEML-PBM on the same data. It is worthy to note that the dinucleotide interactions often appear at TF-binding sites with low binding energy (or information content), please refer to Figure 4C, D, E, and F. Taken together, the new program - BayesPI2 by including dinucleotide energy-dependent model performs well towards the real PBM data.

### Applying BayesPI2 on 66 mouse TFs (good quality versus bad quality)

Both BayesPI2 energy-independent model and dinucleotide energy-dependent model were applied on Z-score transformed and log-normalized probe intensities of 66 training PBMs [1]. The inferred TF-binding energy matrices were then used to evaluate prediction accuracies at 66 testing PBMs from the same DREAM5 challenge [1]. Results are shown in Table 1 and Additional file 1: Table S1 for TFs with good-quality PBMs and those with bad-quality PBMs, respectively. The two groups were classified earlier based on the paired PBMs quality parameters. For BayesPI2 energy-independent model, the median correlation coefficients between testing probe intensities and BayesPI2 predicted intensities are 0.67 and 0.45 in Table 1 and Additional file 1: Table S1, respectively. A two-tailed T-test of correlation coefficients between Table 1 and Additional file 1: Table S1 gives P-value 6.0958 × 10^-12^, which suggests that algorithm testing prediction accuracy from Table 1 (i.e. 34 TFs with good agreement between training and testing PBMs) is significantly better than that from Additional file 1: Table S1 (i.e. 32 TFs with poor agreement between the two PBMs). Similar T-tests were carried out for algorithm performance scores (i.e. Pearson correlation coefficient of probe intensities) of 26 algorithms published by the DREAM5 challenge [1], more than 85% of algorithms show significant better performance at good PBM quality group than that at poor PBM quality group (i.e. 22 and 23 algorithms with T-test P-value <0.01 in Additional file 1: Tables S2 and S3, respectively). Nevertheless, four algorithms seem to have little effect by the quality of PBMs, where k-mer sequence-specific model plus feature selections were used (i.e. Team_k, Team_B, Team_I, and Seed-and-Wobble).

Notable, in Table 1, 34 TFs are spread to almost 14 different DNA-binding domains such as bHLH (2 TFs) and C_2_H_2_ (7 Tfs). However, in Additional file 1: Table S1, 32 TFs just belong to 6 different DNA-binding domains where almost half of them (15 TFs) are C_2_H_2_ DNA-binding domain (Zinc finger protein). It indicates that the poor quality of some PBM experiments (i.e. Additional file 1: Table S1) may be protein domain specific (i.e. Zinc finger protein in Additional file 1: Table S1). Additionally, if we only consider protein domains with more than two TFs from both Table 1 and Additional file 1: Table S1, then there are three protein domains (i.e. around 67%, 67%, and 60% of TFs from Forkhead, Pou + Homeo, and bZIP, respectively) that received a great boost in testing prediction accuracy (i.e. increase in correlation coefficients > 0.05) after using BayesPI2 binding energy-dependent model. In other words, these three protein domains may more frequently encounter base pair interdependency in the DNA-binding sites than that in the other domains.

Results from both Table 1 and Additional file 1: Table S1 reveal that the testing prediction accuracy based on energy-dependent model is very sensitive to the data quality, and the Bayesian method is robust against the data noise. For example, by using the energy-dependent model of BayesPI2 or BEEML-PBM [1], about 14 and 5 TFs show great improvement (increase in Pearson correlation coefficient >0.05) in the testing prediction accuracy, respectively, over that by the simple energy-independent model; for the same test, no improvement was found by FeatureREDUCE. Among the 5 TFs provided by BEEML-PBM, 3 belong to the good-quality PBM group (i.e. TF_27, TF_32, and TF_53; Table 1) where 2 TFs (TF_27 and TF_53) were identified by BayesPI2, and the remaining 2 TFs are in the bad-quality PBM group (i.e. TF_21 and TF_60; Additional file 1: Table S1) where only one TF (TF_21) was recovered by BayesPI2. Thus, by applying various algorithms on the PBMs, the overlap of predictions is poor for TFs with bad-quality data but robust to TFs with good-quality ones. In summary, the better the PBM data quality, the better the testing prediction accuracy, and both the PBM data quality and the TF-binding site interdependency may be protein domain specific. Especially, the good-quality PBM experiments generally benefit more from biophysical modeling of protein-DNA interactions including dinucleotide interactions, than the poor ones from the same computation.

### Predicted TF-binding energy-level versus paired PBM data quality

**Figure 5:**
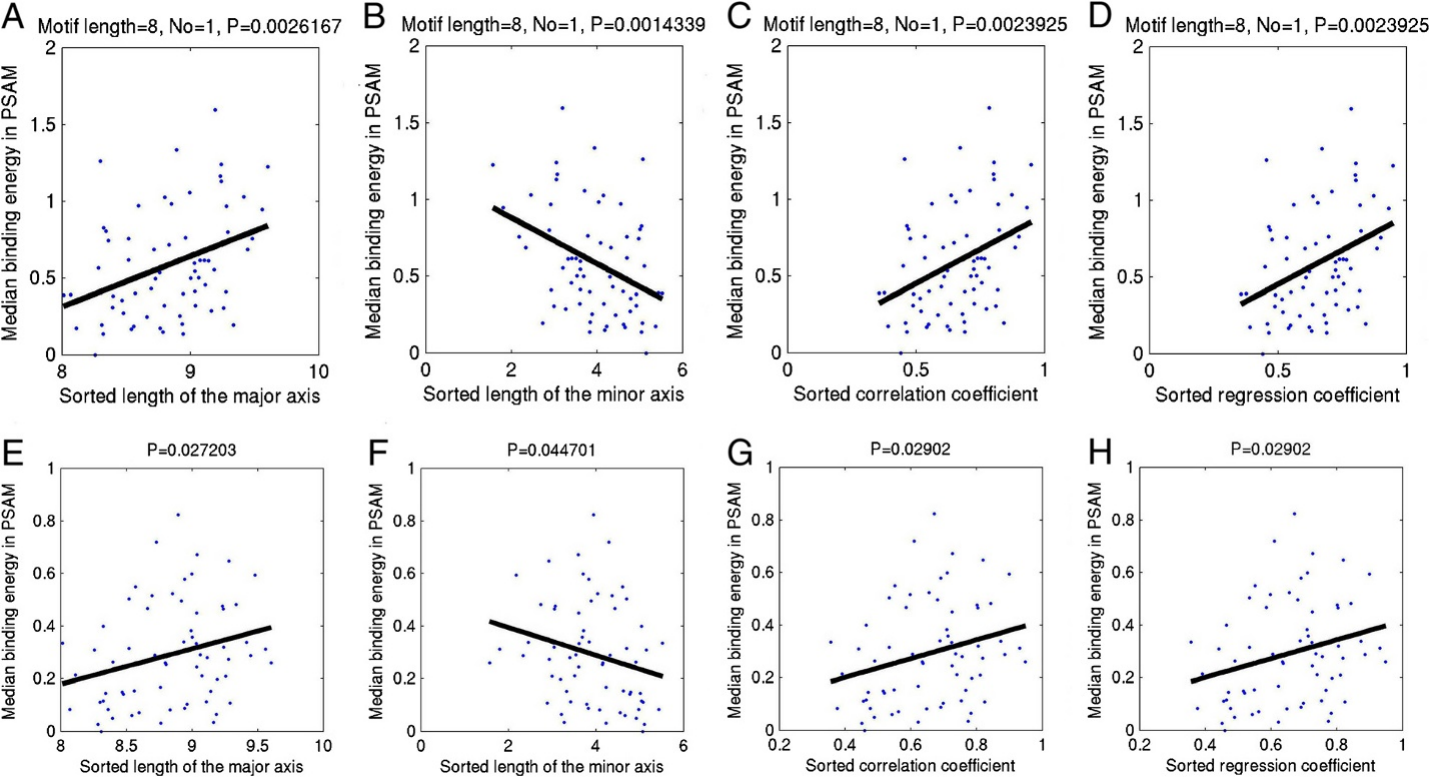
**Scatter plots of predicted binding energy-level in a motif versus sorted PBM quality parameters. A**, **B**, **C**, and **D** are scatter plots of the median binding energy-level (i.e. *E* of a binding energy matrix where *E* < 0) in a motif predicted by BayesPI2 versus sorted length of the major axis of the PCA ellipse, sorted length of the minor axis of the PCA ellipse, sorted correlation coefficients of normalized 8-mer median intensities between paired PBMs, and regression coefficients, respectively. **E**, **F**, **G**, and **H** are scatter plots of the median binding energy-level (i.e.  where *f* is the probability in PWMs and *E* < 0) in a motif provided by the DREAM5 challenge versus sorted length of the major axis of the PCA ellipse, sorted length of the minor axis of the PCA ellipse, sorted correlation coefficients of normalized 8-mer median intensities between paired PBMs, and regression coefficients, respectively. Both PCA ellipse (i.e. 99.73% limit of PCA quality-control ellipses) and regression coefficients are based on scatter plot of normalized 8-mer median intensities between a pair of training and testing PBM experiments. For 66 mouse TFs, the median binding energy-level of a TF is the log-normalized median of the negative energies (i.e. log(-*E*)) in the binding energy matrix (i.e. *E* was the first predicted binding energy matrix by applying BayesPI2 energy-independent model on the probe intensities of PBM training experiments). In the figures, the black smooth line is a fitted linear regression line to the scatter plot, P-value to the regression line is shown at the top of each figure.

Encouraged by the above findings, it is interesting to investigate relationships between the predicted TF-binding energy-level of a motif and the PBM data quality across 66 TFs. First, for each TF, the median of negative binding energies of the first predicted binding energy matrix by BayesPI2 energy-independent model was computed. Then, a log-transformed absolute median energy value was used to summarize the binding energy-level of a motif, which is proportional to the information content of the motif. Scatter plots of the log-transformed median TF-binding energy-level against the sorted paired PBM quality parameters such as the length of the major and minor axes of the PCA ellipse, correlation coefficient and regression coefficient between training and testing PBMs, are displayed in Figure 5A, B, C, and D, respectively. A linear regression line was fitted to every scatter plot, P-value to the regression coefficient shows that the median binding energy-level of a motif is positively correlated to the length of the major axis of the PCA ellipse (P < 0.003) and the correlation coefficients (P < 0.0024), but anti-correlated to the length of the minor axis of the PCA ellipse (P < 0.0015). Results by applying BayesPI2 energy-independent and dinucleotide energy-dependent model (i.e. using either normalized 8-mer median intensities or probe intensities) are available in the (i.e. Additional file 1: Figures S2, S3, S4 and S5), where almost all of them show significant positive correlation between the median binding energy-level of a motif and the paired PBMs data quality, except for few cases (i.e. different motif length) obtained by applying BayesPI2 dinucleotide energy-dependent model on the PBM probe intensities. Hence, the better the PBM data quality, the higher the binding energy-level (or information content) of a motif.

To verify the present finding, the same analysis was performed again on a set of PWMs provided by the DREAM5 challenge [1], where the PWMs were predicted by various methods based on the same training PBM experiments for 66 mouse TFs. Methods that were used to obtain those PWMs include both biophysical free-energy models (i.e. BEEML-PBM [22], FeatureREDUCE, and MatrixREDUCE [11]) and other model types (i.e. PWM + HWMs from Team_E [1], and RankMotif [23]). First, each PWM was converted to TF-binding energy matrix. Then, scatter plots of log-transformed median binding energy-level of a PWM versus sorted paired PBM quality parameters were made (i.e. Figure 5E, F, G, and H). P-values of regression coefficients to the length of the major and minor axes of the PCA ellipse, and the correlation coefficients are P < 0.03, P < 0.045, and P < 0.03, respectively. It is clear that there is a strong positive correlation between the predicted binding energy-level of a motif and the PBM data quality. Overall, the results of published PWMs are consistent with the previous findings by using the BayesPI2 predicted binding energy matrices. It reinforces the hypothesis that methods for modeling TF sequence specificity are extremely sensitive to the PBM data quality. Specifically, the low energy-level (information content) of a predicted binding energy matrix may be caused by the poor PBM data quality (i.e. the poor agreement between training and testing PBMs).

### Algorithm performance comparison and verification of predicted PBEM in ChIP-seq data

A comparison of algorithm performance between the BayesPI2 and the other methods was carried out, where the median Pearson correlation coefficients (i.e. correlation between the predicted probe intensities and the actual intensities) of 66 mouse TFs were computed for each algorithm (Additional file 1: Table S4). The correlation coefficients based on bayesPI2 and 26 other algorithms were obtained from this study (i.e. Table 1 and Additional file 1: Table S1) and the earlier publication [1] (i.e. Supplementary Table 3 of original paper), respectively. A scatter plot of the sorted median Pearson correlation coefficients for all algorithms is shown in Additional file 1: Figure S6 where the performance of BayesPI2 is close to the top 10 ranked algorithms from the original paper. However, it should be noted that the present comparison may not tell the true merit of each algorithm because of the poor data quality in PBM experiments (i.e. Additional file 2 versus supplementary Figure 4 of original paper). For computational cost, BayesPI2 takes ~7 min and ~30 min to predict one PBEM (i.e. using ~40000 PBM probe sequences and ~600 MB memory) at a Linux cluster machine by applying energy-independent model and energy-dependent models, respectively. However, for BEEML-PBM, the same computer could not complete the prediction of one PBEM (i.e. using the same input data) including dinucleotide interaction energies after running for almost 14 days with ~12 GB memory. Taken together, the new program BayesPI2 is an efficient and robust tool to analyze large data sets such as PBM.

**Figure 6:**
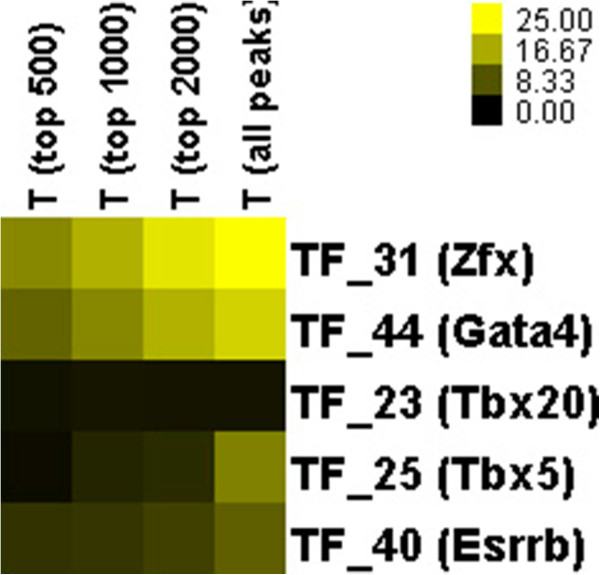
**Heat map of t-values obtained from the linear regression analysis of dependence between the inferred TF-binding affinities and the measured TF tag counts in ChIP-seq experiment.** For 5 mouse TFs with available *in vivo* ChIP-seq data sets, the relationships between the predicted TF-binding affinities by using PBEM from *in vitro* data and the measured TF occupancy data from *in vivo* ChIP-seq experiments were investigated by the linear regression model, respectively. In the figure, t-value represents the significance of such correlations. The top 500, 1000, 2000, and all called peaks were considered in the linear regression analysis, respectively. Here, the top-ranked peaks were sorted in descending order by the number of tags found at the peak.

Subsequently, PBEMs of five mouse TFs obtained by BayesPI2 based on *in vitro* data were used to predict TF occupancy data in the corresponding *in vivo* ChIP-seq data [1]. Among the five mouse TFs, three (i.e. TF_31 Zfx; TF_44 Gata4; TF_23 Tbx20) were classified as good-quality PBMs (Table 1) and the other two (i.e. TF_25 Tbx5; TF_40 Esrrb) were defined as poor PBMs (Additional file 1: Table S1) in this study. Results are shown in Table 2 (square root of R-square statistic) and Figure 6 (T values of t-statistic), where a linear regression model was used to evaluate the significance of dependence between the inferred PBEM from *in vitro* data and the measured TF tag counts from ChIP-seq experiment. For every *in vivo* data, the analysis was done at the top 500, 1000, 2000 ranked peaks (i.e. sorted by the number of tags found at the peak, in descending order), and all called peaks, respectively. In each selection of the top-ranked peaks, the same amount of bottom-ranked peaks was also considered by the regression analysis. The results tell that there are significant correlations between the predicted TF-binding affinities and the measured tag counts across different sizes of input peaks, by using inferred PBEMs from two TFs with good PBM data quality (i.e. TF_31 and TF_44). However, for the other two TFs (TF_25 and TF_40) having poor PBM data quality, the significance of such dependence is weak and different among various sizes of input data. Especially, both R-square statistic and T-values obtained from TFs with bad-quality PBMs are much smaller than those provided by the good-quality ones. Thus, poor PBM data quality may result in unreliable prediction of PBEM (i.e. algorithms may learn the background signals), which hinders any subsequent genomic analysis. Consequently, it leads to poor agreement between the estimated TF-binding affinities based on the PBEM and the measured TF occupancy data from the *in vivo* data.

**Table 2:** **Correlations between the estimated TF-binding affinities based on inferred PBEM from** ***in vitro*** **data and the measured TF tag counts from** ***in vivo*** **ChIP-seq experiment**

ChIP-Seq	Called peaks	Rank	Paired PBMs agreement	R (top 500)	R (top 1000)	R (top 2000)	R (all peaks)
TF_31 (Zfx)	10338	8	Good	0.38978	0.35943	0.33566	0.25502
TF_44 (Gata4)	16979	22	Good	0.29675	0.28458	0.2645	0.15614
TF_23 (Tbx20)	4012	33	Good	0.077781	0.062428	0.043291	0.043338
TF_25 (Tbx5)	56352	41	Bad	0.062865	0.090766	0.073079	0.053016
TF_40 (Esrrb)	21647	59	Bad	0.16334	0.12283	0.10351	0.064026

In the table, the first column describes ID of mouse TFs in DREAM5 challenges and the TF names to the ChIP-seq experiments; Called peaks are the number of called peaks in the ChIP-seq data; Rank represents rank order of TFs that were sorted in decreasing order of the final performance score across all tested algorithms in Figure 2 of original publication [1]; paired PBMs agreement indicates the agreement between training and testing PBMs; R is square root of R-square statistic from a linear regression model, where the relationship between the predicted TF-binding affinities and the observed ChIP-seq tag counts is investigated.

## Discussion

In this work, new quality-control parameters (i.e. PCA ellipse) were developed to assess the quality of PBM. Both single and paired PBM data quality can be illustrated in a scatter plot, where predefined control limit (i.e. ) by PCA quality-control ellipse gives a direct assessment of measurement attribute. For example, the lengths of the major and minor axes of the PCA ellipse represent the dynamical range of PBM signal intensities, and the overall difference between paired PBMs, respectively. For single PBM, algorithm performance at the testing data may be predicted for ~70% of TFs based on the corresponding training data quality. For paired PBMs, a visual inspection of PCA quality-control ellipse on the scatter plot can not only identify data outliers but also tell the robustness of agreement between the two observations. On the contrary, correlation coefficient is easily affected by the data outliers (Additional file 1: Figure S7), which is not suited to measure agreement between paired observations [20]. Based on the estimated PBM quality information for 66 mouse TFs from the DREAM5 challenge, several interesting findings were revealed: for instance, both training data quality and paired PBMs agreement (Figures 2 and 3) are significantly correlated to the TF rank order according to Figure 2 of original publication [1], where 66 TFs were sorted in decreasing order by the mean of final algorithm performance scores. The results indicate that the decrease in algorithm performances across 66 mouse TFs in the DREAM5 challenge is mostly due to the gradual reduction of PBM data quality, especially the poor agreement between training and testing PBMs.

In addition to the new PBM quality-control parameters, a biophysical model of protein-DNA interactions including adjacent dinucleotide interdependence was newly implemented in C – BayesPI2, where sparse Bayesian learning approach was used to infer free-energy model parameters. The new energy-dependent model is able to recover known nucleotide interdependent effects on the binding affinities for Egr1 and Hnf4a, respectively. The results also reveal that dinucleotide interdependence often occurs at low binding energy (or information content) sites (Figure 4), which are significantly influenced by PBM data quality. Particularly, the new BayesPI2 dinucleotide energy-dependent model offers great improvement in testing prediction accuracy over the simple energy-independent model, for at least 21% of the analyzed mouse TFs (i.e. Table 1 and Additional file 1: Table S1). The new improvement might have resulted from more motif lengths searched by this study. Alternatively, the over-fitting data problem, which hampers regression-based free-energy model [1, 19] to estimate a large number of unknown model parameters (i.e. BEEML-PBM in R), is minimized by the Bayesian implementation of nonlinear parameter fitting.

Equipped with both the new quality-control parameters for paired PBMs and the new free-energy model including dinucleotide interdependence, 66 mouse TFs from the DREAM5 challenge were first classified into two groups (i.e. good-quality and bad-quality PBMs in Table 1 and Additional file 1: Table S1, respectively) based on the agreement between training and testing PBMs, then the algorithm accuracy on the test sets and the improvement by the energy-dependent model over the simple energy-independent model were compared between the two groups. Four new observations were revealed by this work: 1) the algorithm testing accuracy at good-quality PBMs is significantly better (P > 7 × 10^-12^) than that at the bad-quality ones (Additional file 1: Tables S2 and S3); 2) the poor PBM data quality is protein domain specific because almost half of the bad-quality PBMs (Additional file 1: Table S1) belong to C_2_H_2_ DNA-binding domain (Zinc finger protein); 3) the improvement in algorithm testing prediction accuracy by using the energy-dependent model over that by the simple energy-independent model is not only associated with the PBM data quality, but also linked to the specific protein domains (i.e. Forkhead, Pou + Homeo, and bZIP); 4) the predicted binding energy-level (or information content) of a motif is significantly correlated to the quality of paired PBMs (i.e. the better paired the PBM data quality, the higher the predicted binding energy-level; Figure 5).

From these four new discoveries, two (i.e. the better the PBM quality, the better the prediction accuracy; and the better the PBM quality, the higher the binding energy-level of a motif) were observed in both the BayesPI2 predictions and the original results from the DREAM5 challenge [1]. Particularly, the predicted PBEMs from the good-quality PBMs (i.e. TF_31 and TF_44) performed significantly better than those inferred by the poor PBMs (i.e. TF_25 and TF_40), on subsequent genomic analysis in *in vivo* data such as ChIP-seq. Nevertheless, there is a poor correlation between the inferred TF-binding affinities and measured ChIP-seq signals for TF_23 (TBX20), a putative good-quality PBM in Table 2 and Figure 6. This may be caused by the limitation of *in vivo* experiment. For example, ChIP-seq may not necessarily identify the direct TF-DNA interactions [24]), and TBX20 is known to directly interact with a number of proteins in regulation of gene expression [25]. The remaining two findings are supported by the literature evidences: 1) for protein domain specific PBM data quality, it is known [21] that binding of C_2_H_2_ zinc finger proteins are often not well measured in PBM experiments because many C_2_H_2_ proteins do not bind specific DNA sequences in PBM experiments; 2) for protein domain specific dinucleotide interdependence, two of the protein domains (i.e. Pou + Homeo and bZIP) were known to contain nucleotide dependence at the binding sites [21] (i.e. the homeodomain recognition helix is associated to base pair interdependency to DNA-binding, and many bZIP factors frequently bind to two distinct half-sites that may result in dinucleotide interaction). Therefore, both PBM data quality and computational modeling of protein-DNA interactions are influenced by the specific protein domains, and certain protein domains may require a free-energy model including dinucleotide interdependence to obtain precise binding energy matrix. It is important to note that the associations of both the PBM data quality and the dinucleotide energy-dependence with the protein structure classes are only revealed by this study, after applying the new PBM quality-control parameters and BayesPI2.

To minimize the effect of PBM data quality on downstream data analysis, DNA microarray experiment design from the previous works [26] might be introduced. Especially, triplet PBM experiments may be better than paired PBMs design to distinguish the experimental failure (i.e. a poor PBM data quality) from the biological failure (i.e. the TF does not bind to a DNA sequence). An alternative computational solution, to the PBM data quality issue, is to integrate k-mer sequence specific model plus feature selections into the PWM energy model (i.e. the top-ranked algorithm – FeatureREDUCE in the original study [1]). That is because k-mer sequence specific model (i.e. Teams K, B, I, and Seed-and-Wobble; uses the highest-affinity k-mer) does not consider the intensity values of PBMs when learning the motif. It is robust against the data noise that may provide a good initial seed motif for regression-based free-energy model to estimate a precise PBEM.

## Conclusion

In conclusion, both the new PBM quality-control parameters and the new biophysical modeling of TF-DNA interactions including dinucleotide interdependence are developed during this work. By applying both methods on paired PBMs for 66 mouse TFs from the DREAM5 challenge, we found that: Bayesian method is robust against the data noise, and mononucleotide PWM methods do not perform similarly to more advanced dinucleotide PWM algorithms for modeling TF sequence specificity. For instance, the BayesPI2 energy-dependent model offers great improvement, for ~21% of the examined TFs, in the testing prediction accuracy over that by the simple energy-independent model. Especially, the PBM data quality not only impacts the algorithm performance, but also influences the inferred binding energy-level of a motif (e.g. the better the PBM data quality, the higher the inferred binding energy-level (information content)). This work may help tremendously for future research in developing computational methods and designing PBM experiments.

## Methods

### Principal component analysis – quality control ellipse

To check the agreement of two measurements by principal component analysis (PCA) [27], sample mean, sample variance, and the covariance between two observations are needed. Let X_1_ and X_2_ be two observations (i.e. the normalized 8-mer median intensities of a mouse TF) under experiments one (i.e. training PBM) and two (i.e. testing PBM), respectively. *X* = [*X*_1_, *X*_2_] are two n × 1 vectors where n is the number of observations in the experiments. The vectors of sample mean are  and the sample covariance matrix is . By performing PCA on the covariance matrix S, principal component coefficients are obtained: for example, *U*^'^*SU* = *L* where *U* and *L* are the eigenvectors (characteristic vectors; ) and eigenvalues (characteristic roots; ) of S, respectively. Then, the *n* variables *X* (i.e. the vector of *X*_*1*_ and *X*_*2*_) are transformed to *n* uncorrelated principal components *Z* based on equation . Subsequently, *Z* is scaled to y-score,  with unit variance. Finally, T-score to a pair of observations can be computed by *T*^2^ = *Diag*(*Y*^'^*Y*) where *Diag* is the main diagonal of *Y*^'^*Y*. It is an overall measurement of the conformance of an observation to its mean. Thus, the quantity *T*^*2*^ gives a direct assessment of similarity between two measurements. Any observation vectors that produce values of *T*^*2*^ greater than a predefined threshold  will be out of control in a quality control (or PCA) ellipse. To compute the quality-control limit , a probability value *p* needs to be defined. For example, in this work, the limit of quality-control ellipse is three times the standard deviation (~99.73%) from sample mean, then the probability value *p* is ~0.0027 and the  is computed from F distribution [28] . To construct a unique quality-control ellipse in the two-dimensional case, the length of the major and minor axes, their orientation, and their interaction are needed. These information can be easily obtained from the length of the semi-major () and semi-minor () axes, the slope of the major (*V*_*21*_*/V*_*11*_) and minor (-*V*_*11*_*/V*_*21*_) axes where *V* = *UL*^1/2^, and the major and minor axes of the ellipse intersect at the sample mean .

### Quality control parameters for PBM

Two types of quality-control parameters are defined for PBM: one is single PBM data quality and the other is paired PBM data quality. For the former, correlation coefficient between normalized signal intensities and normalized background intensities, the length of the major and minor axes of PCA ellipse, and regression coefficient are considered. Both PCA ellipse and regression coefficient were based on an M versus A scatter plot, where . For the second one, it includes the length of the major and minor axes of PCA ellipse, correlation coefficient of normalized 8-mer median intensities between a pair of training and testing PBM experiments, and regression coefficient. Here, both PCA ellipse and regression coefficient are obtained from a scatter plot of normalized 8-mer median intensities between a pair of PBMs. In the scatter plot, the orientation and the length of the major PCA ellipse axis is related to the first principal component, which represents the dynamical range of PBM signal intensities (i.e. with 99.73% limit of PCA quality control ellipse, ~99.73% of measured signals are included in the quality ellipse); the orientation and the length of the minor PCA ellipse axis is the second principal component, which shows disagreements between the two observations. Generally, if there is good agreement between two PBMs, then a narrow PCA ellipse is obtained [4], which means that both PBMs are of good quality. If the PCA ellipse is wide, then the observations of the two PBMs are in poor agreement, which indicates that data quality of one of the PBMs is in question. Subsequently, the algorithm testing prediction accuracy may not be reliable due to poor agreement between training and testing PBM experiments. Taken together, the length of the major axis of the PCA ellipse (i.e. the dynamical range of PBM signal intensities) is an essential quality-control parameter for both signal and paired PBMs.

### Biophysical model of protein-DNA interaction including dinucleotide interdependence

A biophysical free-energy model of TF-DNA interactions [5, 12] is adopted in this work, where a Fermi-Dirac form of TF-binding probability *P(S)* is used to estimate TF-binding energy *E(S)* to a short stretch of DNA sequence *S*. For a detailed description of the biophysical model of protein-DNA binding, please refer to previous papers [12, 29]. Here, the binding probability is defined by


where *E(S)* is the TF-binding free energy, and *μ* is the chemical potential set by the TF concentration. Please note that negative binding energy is often interpreted as the information content used by information-based weight matrix [30]. In the new BayesPI2 program, sequence-specific interaction is included in the TF-binding energy


where *E*_*i*,*a*_ is the interaction energy with nucleotide *a* ∈ (*A*, *C*, *G*, *T*) at position *i* = 1, 2, … *L* of the DNA sequence; *S*_*i*,*a*_ characterizes the sequence, *S*_*i*,*a*_ = 1 if *i*-th bases is *a*, otherwise *S*_*i*,*a*_ = 0;  is a pair of dependent energy correction (i.e. *a* at position *i*, *b* at position *j, a*, *b* ∈ (*A*, *C*, *G*, *T*) ). In the calculation, only adjacent nucleotide interactions are considered in , which reduces the number of pair-dependent energy corrections from 16*L*^2^ to 16(*L* - 1), *L* is the length of a motif. This simplified version of TF-DNA interaction including dinucleotide interdependence was used by an earlier paper [22] too.

### Sparse Bayesian learning of model parameters

Based on the previous papers [12, 29], a new Bayesian neural network framework to infer free-energy model parameters is developed. For example, to minimize errors between predicted TF-binding affinity and measured TF-binding signals, the objective function is


where  and  are the model error function and model regularization function, respectively; *T*_*i*_ is the *i*th measured TF-binding signal and *Y*_*i*_ is the predicted TF occupancy according to the protein-binding probability *P*(*S*); α and β are the two unknown hyperparameters that control the model parameters and the data noise level, which are determined by the input data; and *w*, *Λ*, *Γ* represent the model parameters, the input data, and the hypothesis models (i.e. the protein-binding probability and the regularization function), respectively. After adding dinucleotide interdependence term into the protein-binding probability *P*(*S*), far more model parameters need to be trained by the input data than that of an independent free-energy model [12]. To avoid over-fitting problem that may be caused by a large number of unknown model parameters, an approach similar to Bayesian sparse learning and the relevance vector machine [19] is used. For instance, the term *α* · *E*_*w*_ is divided into several distinct groups


where *w*_1_ and *b*_1_ are the output layer parameters of neural networks, and *μ*, *E*_*i*,*a*_, and  represent the chemical potential, the TF-binding energy matrix and the dinucleotide-dependent energy correction, respectively. If *α*_*i*_ is large, then the model parameters are close to zero, which are not important for the minimization function. Conversely, if the *α*_*i*_ is small, then the corresponding model parameters are important for the data fitting. In this way, sparse Bayesian learning of model parameters can be realized. Following the new Bayesian parameter minimization framework, a set of new update functions (i.e. back-propagation neural networks, Bayesian evidence approximation with R-propagation algorithm) [12] were derived and implemented in BayesPI2, a new C program for inferring protein-DNA binding energy matrix.

### Unsupervised classification of 66 mouse TFs by PBM quality-control parameters

An in-house made fuzzy neural gas algorithm, a combination of fuzzy logic and neural gas algorithm, was used to classify 66 TFs into two classes based on PBM quality-control parameters. The neural gas algorithm uses a similar “soft-max” adaptation rule as maximum-entropy clustering and self-organizing maps (SOMs) to summarize high-dimensional input space (i.e. TFs) to low-dimensional reference vector space (i.e. class prototype). Then, the TF is assigned to the nearest class prototype and the fuzzy membership estimates the confidence level of the classification. Though many other machine-learning methods can be utilized to perform the same task, the Fuzzy neural gas algorithm is capable of performing unsupervised learning and capturing nonlinear relationships between the features (i.e. quality-control parameters) and sample classes [31].

### PBM experiments

PBM of mouse TFs Egr1 and Hnf4a were downloaded from UniProbe database [32]. For both TFs, one of the replicate PBM experiments was used as training set and the other was used as test set to verify the newly developed BayesPI2 including dinucleotide interdependence. Both training and testing PBM experiments of 66 mouse TFs were obtained from the DREAM5 challenge [1], where normalized raw probe intensities were used to evaluate algorithm testing performance and single PBM data quality, and normalized 8-mer median intensities were used to evaluate paired PBM data quality. For all calculations, the normalization of PBM data is based on Z-score transformation of log-normalized signal intensities.

### ChIP-seq data

To evaluate the predicated PBEM on *in vivo* data, ChIP-seq data sets of five mouse TFs were obtained: Esrrb (TF_40; GEO accession GSM288355), Zfx (TF_31; GEO accession GSM288352), Tbx20 (TF_23; GEO accession GSM734426), Tbx5 (TF_25; GEO accession GSM558908), and Gata4 (TF_44; GEO accession GSM558904). For each *in vivo* data set, the predicted PBEM by BayesPI2 from *in vitro* PBM experiment was used to scan DNA sequences of all called peaks, where the TF-binding affinities were computed based on the middle 200 bases of each peak. The affinity-based analysis of DNA sequences is similar to MatrixREDUCE [33] but Fermi-Dirac form of TF-binding probability is used by BayesPI2. Then, a linear regression model is applied to test the dependence between the estimated TF-binding affinity by using *in vitro* PBEM and the observed TF tag counts from *in vivo* data. For example, the ChIP-seq tag counts represent response variables and the estimated TF-binding affinities are explanatory variables in a linear regression model. Finally, the T values and the correlation coefficients from the linear regression analysis are used to evaluate the significance of correlations between the estimated TF-binding affinities and the actual TF occupancy data.

### Computer programs

Inferred PBEMs from PBM experiments for 66 mouse TFs by using BayesPI2, C version of BayesPI2 program, and MATLAB program of PCA quality control ellipse are publically available http://folk.uio.no/junbaiw/CBayesPI2

## Electronic supplementary material

Additional file 1: **The file contains supplementary results, figures, tables, and a description of data and code etc.** (DOC 2 MB)

Additional file 2: **The file contains figures of predicted binding energy matrices of 66 mouse TFs from DREAM5 challenge by using BayesPI2 energy-independent model.** The order of figures are the same as Additional file 1: Figure S4. of original publication [1]. (ZIP 13 MB)
