# Supplementary material for: Quality versus accuracy: result of a reanalysis of protein-binding microarrays from the DREAM5 challenge by using BayesPI2 including dinucleotide interdependence
Source: BMC Bioinformatics. 2014 Aug 27;15(1):289. doi: 10.1186/1471-2105-15-289 (PMC4161872; doi:10.1186/1471-2105-15-289)
Supplement: Supplementary file 1 — Additional file 1: The file contains supplementary results, figures, tables, and a description of data and code etc. (DOC 2 MB) [file 12859_2014_6559_MOESM1_ESM.doc]

**Supplementary “Quality verse accuracy: Result of a reanalysis of protein-binding microarrays from the DREAM 5 challenge by BayesPI2 including dinucleotide interdependence”**

**Junbai Wang**

**Supplementary Figures**

**S-Figure 1. Scatter plots of algorithm performance rank order verse PBM testing data quality.**


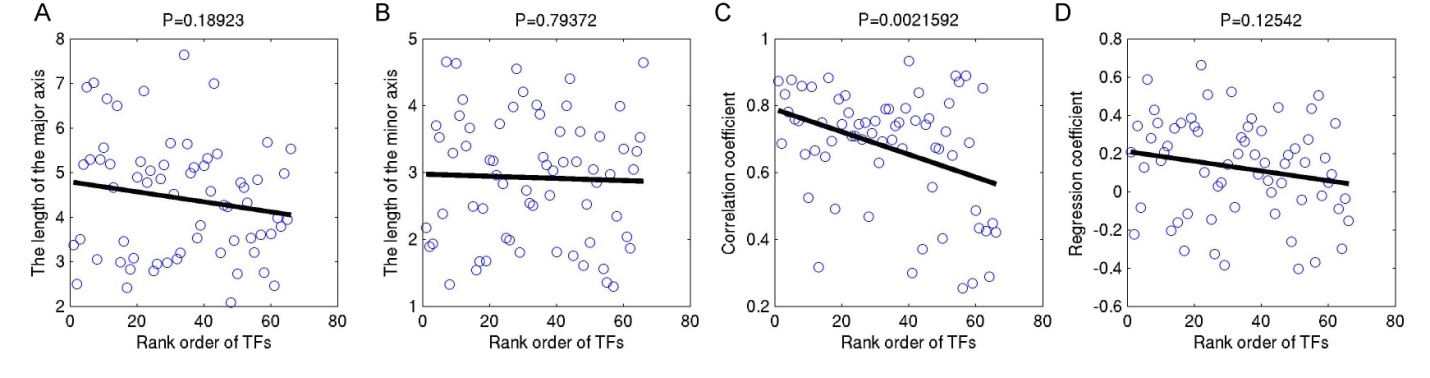


Figures S1-A, S1-B, S1-C and S1-D show scatter plots of algorithm performance rank order of 66 mouse TFs verse the length of the major axis of the PCA ellipse (i.e. 99.73% limit of PCA quality control ellipse), the length of the minor axis of the PCA ellipse, correlation coefficient between signal intensities and background intensities, and regression coefficients, respectively. Both PCA ellipse and the regression coefficient are based on MA scatter plots of testing PBM experiments. In the figure, the black smooth lines are fitted linear regression lines to the scatter plots, and the P-values of the regression lines are indicated at the top of each figure. The rank order of TFs (i.e. 66 TFs are sorted in decreasing order by mean final algorithm performance scores) is adopted from the Figure 2 of the DREAM5 challenge publication.

**S-Figure 2. Scatter plots of predicted binding energy level in a motif verse sorted paired PBM quality parameters (8-mer intensity with BayesPI2 energy-independent model).**


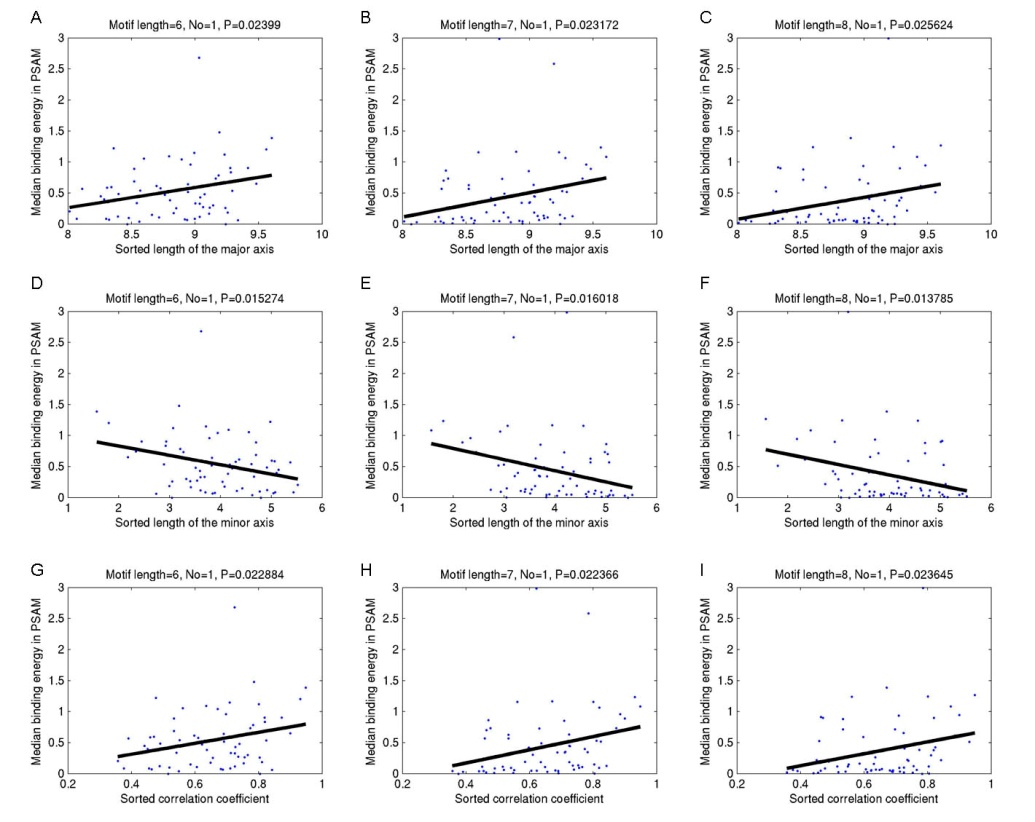


Figures S2-A, S2-B and S2-C are scatter plots of the median binding energy level of a motif verse sorted length of the major axis of the PCA ellipse for motif length 6, 7, and 8, respectively. Figures S2-D, S2-E and S2-F are scatter plots of the median binding energy level of a motif verse sorted length of the minor axis of the PCA ellipse for motif length 6, 7, and 8, respectively. Figures S2-G, S2-H and S2-I are scatter plots of the median binding energy level of a motif verse sorted correlation coefficients of 8-mer median intensities between training and testing PBM experiments for motif length 6, 7, and 8, respectively. The PCA ellipse (i.e 99.73% limit of PCA quality control ellipses) is based on scatter plot of 8-mer median intensities between a pair of training and testing PBM experiments. For 66 TFs, the median binding energy level of each TF is the log normalized median of negative binding energies (i.e.$\log\left(-E\right)$ where$E<0$) in the first predicted binding energy matrix, by applying BayesPI2 energy-independent model on normalized 8-mer median intensities of training PBM experiments. In the figure, the black smooth lines are the fitted linear regression lines to the scatter plots, P-values to the regression lines are shown at the top of each figure.

**S-Figure 3.** **Scatter plots of predicted binding energy level in a motif verse sorted paired PBM quality parameters (8-mer intensity with BayesPI2 energy-dependent model).**


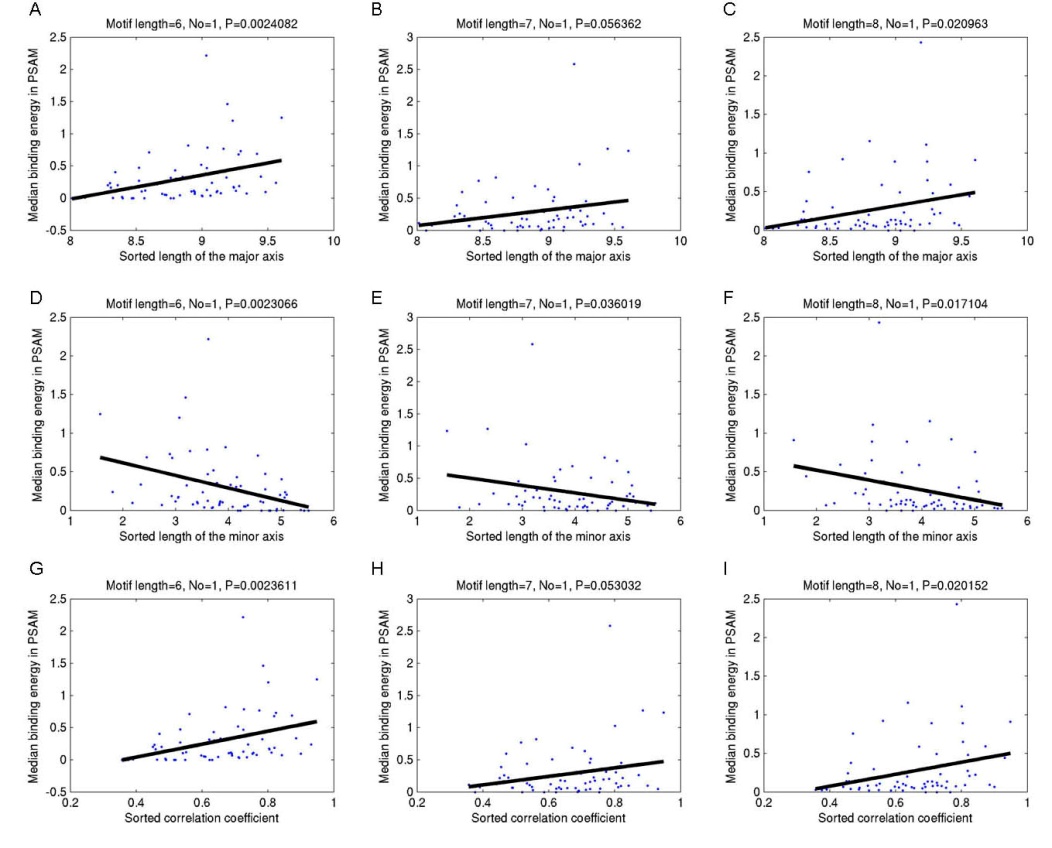


Figures S3-A, S3-B and S3-C are scatter plots of the median binding energy level of a motif verse sorted length of the major axis of the PCA ellipse for motif length 6, 7, and 8, respectively. Figures S3-D, S3-E and S3-F are scatter plots of the median binding energy level of a motif verse sorted length of the minor axis of the PCA ellipse for motif length 6, 7, and 8, respectively. Figures S3-G, S3-H and S3-I are scatter plots of the median binding energy level of a motif verse sorted correlation coefficients of 8-mer median intensities between training and testing PBM experiments for motif length 6, 7, and 8, respectively. The PCA ellipse (i.e 99.73% limit of PCA quality control ellipses) is based on scatter plot of 8-mer intensities between a pair of training and testing PBM experiments. For 66 TFs, the median binding energy level of each TF is the log normalized median negative energies (i.e.$\log\left(-E\right)$ where $E<0$) in the first predicted binding energy matrix, by applying BayesPI2 energy-dependent model on normalized 8-mer median intensities of training PBM experiments. In the figure, the black smooth lines are fitted linear regression lines to the scatter plots, P-values to the regression lines are shown at the top of each figure.

**S-Figure 4.** **Scatter plots of predicted binding energy level in a motif verse sorted paired PBM quality parameters (raw probe intensity with BayesPI2 energy-independent model).**


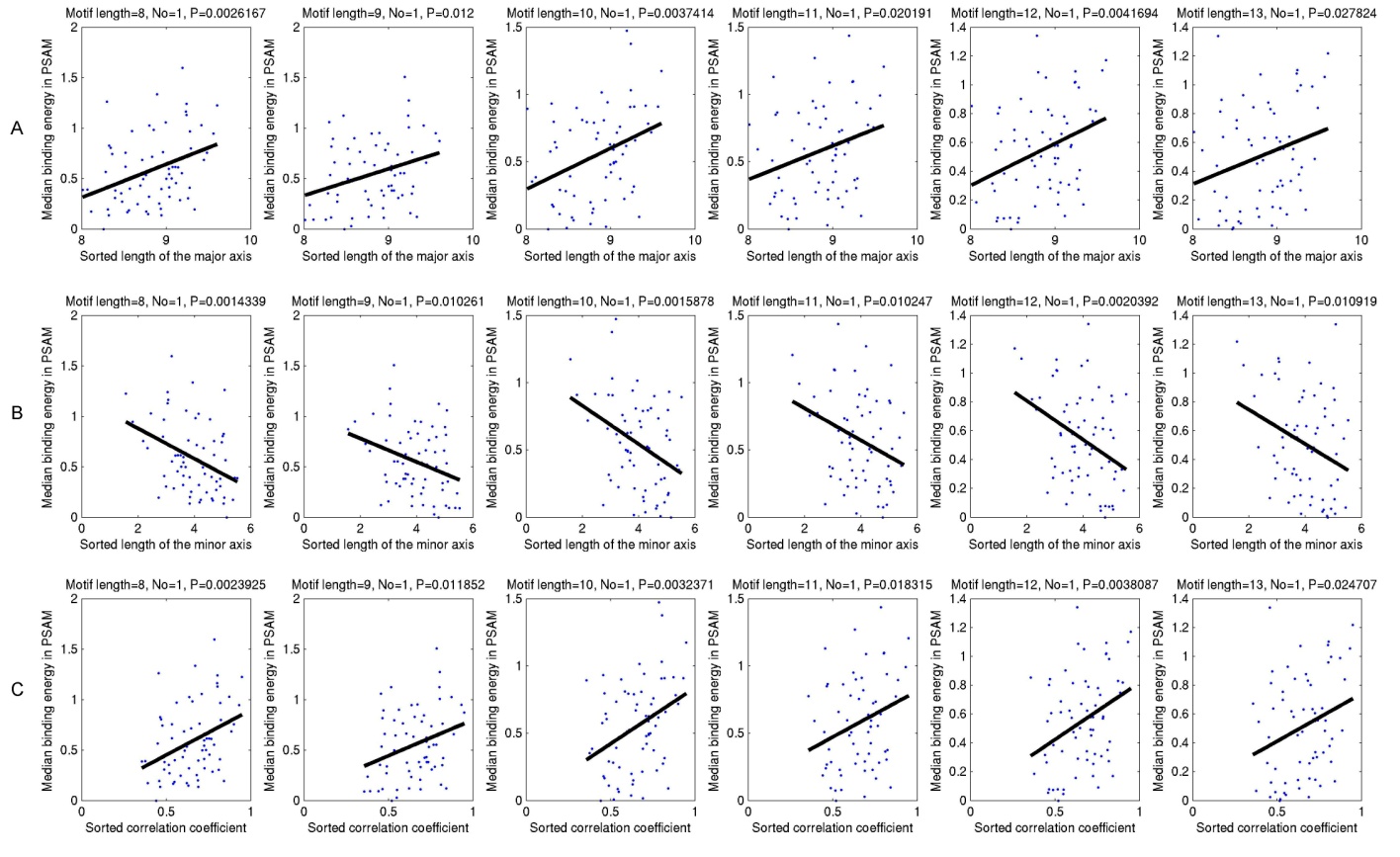


Figure S4-A are scatter plots of the median binding energy level of a motif verse sorted length of the major axis of the PCA ellipse for motif length 8, 9, 10, 11, 12 and 13, respectively. Figures S4-B are scatter plots of the median binding energy level of a motif verse sorted length of the minor axis of the PCA ellipse for motif length 8, 9, 10, 11, 12 and 13, respectively. Figures S4-C are scatter plots of the median binding energy level of a motif verse sorted correlation coefficients of 8-mer median intensities between training and testing PBM experiments for motif length 8, 9, 10, 11, 12 and 13, respectively. The PCA ellipse (i.e 99.73% limit of PCA quality control ellipses) is based on scatter plot of 8-mer median intensities between a pair of training and testing PBM experiments. For 66 TFs, the median binding energy level of each TF is the log normalized median negative energies (i.e.$\log\left(-E\right)$ where$E<0$) in the first predicted binding energy matrix, by applying BayesPI2 energy-independent model on normalized probe intensities of training PBM experiments. In the figure, the black smooth lines are the fitted linear regression lines to the scatter plots, P-values to the regression lines are shown at the top of each figure.

**S-Figure 5.**  **Scatter plots of predicted binding energy level in a motif verse sorted paired PBM quality parameters (raw probe intensity with BayesPI2 energy-dependent model).**


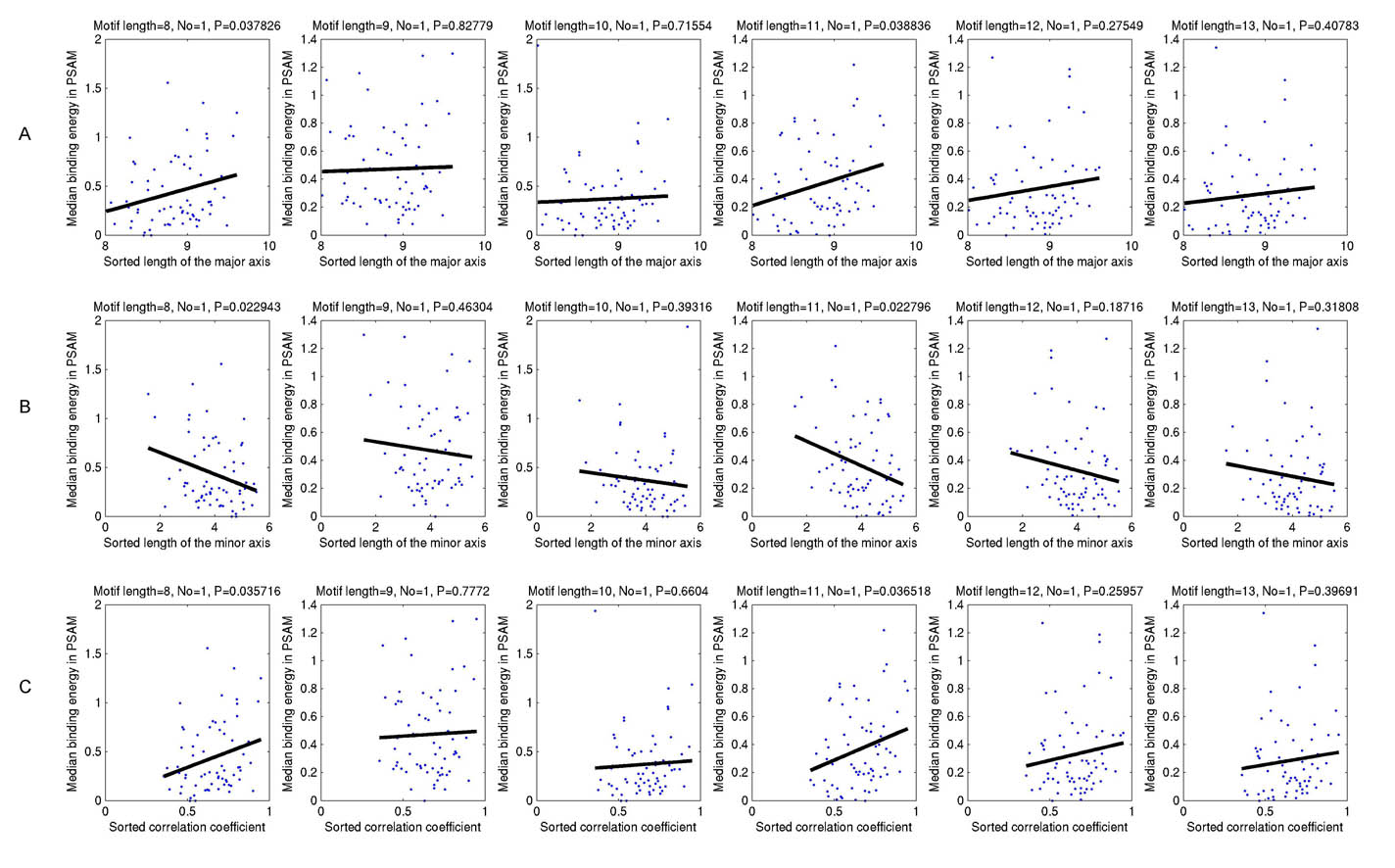


Figure S5-A are scatter plots of the median binding energy level of a motif verse sorted length of the major axis of the PCA ellipse for motif length 8, 9, 10, 11, 12 and 13, respectively. Figures S5-B are scatter plots of the median binding energy level of a motif verse sorted length of the minor axis of the PCA ellipse for motif length 8, 9, 10, 11, 12 and 13, respectively. Figures S5-C are scatter plots of the median binding energy level of a motif verse sorted correlation coefficients of 8-mer median intensities between training and testing PBM experiments for motif length 8, 9, 10, 11, 12 and 13, respectively. The PCA ellipse (i.e 99.73% limit of PCA quality control ellipses) is based on scatter plot of normalized 8-mer median intensities between a pair of training and testing PBM experiments. For 66 TFs, the median binding energy level of each TF is the log normalized median negative energies (i.e.$\log\left(-E\right)$ where$E<0$) in the first predicted binding energy matrix, by applying BayesPI2 energy-dependent model on normalized probe intensities of training PBM experiments. In the figure, the black smooth lines are fitted linear regression lines to the scatter plots, P-values to the regression lines are shown at the top of each figure.

**S-Figure 6. Algorithms performance comparison between BayesPI2 and 26 published algorithms from DREAM5 challenges.**


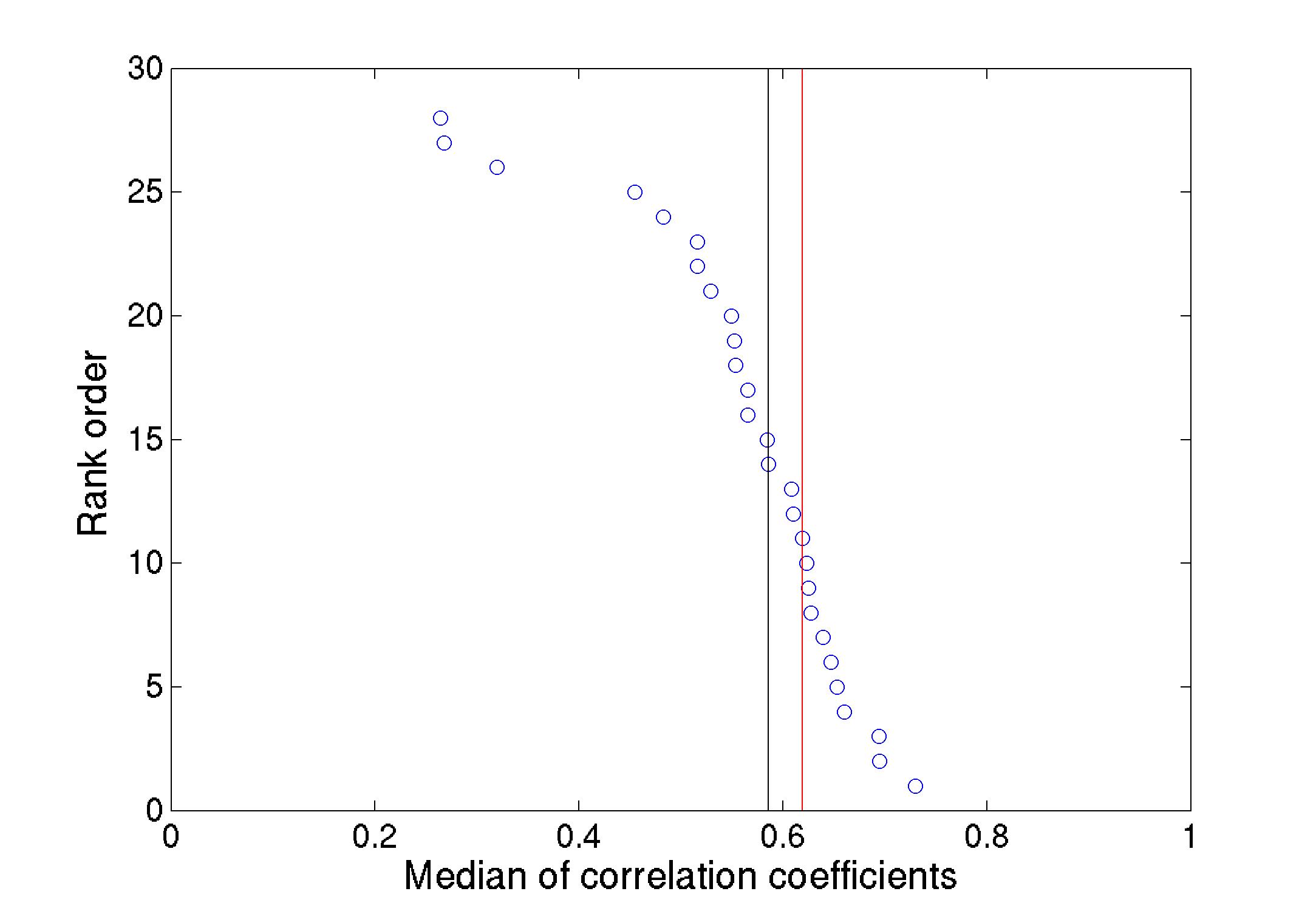


The median Pearson correlation coefficients (i.e. correlations between the predicted probe intensities and the actual PBM intensities for 66 mouse TFs, respectively) of all tested algorithms were sorted in descending order in X-axis, and the corresponding rank order is shown in the Y-axis. The Red and black vertical lines represent the position of median correlation coefficient to BayesPI2 energy-dependent model and BayesPI2 energy-independent model, respectively. Here correlation coefficients of 66 mouse TFs for BayesPI2 and 26 algorithms were obtained from the current study (Table 1 and STable 1) and the supplementary Table 3 of earlier publication [1], respectively.

**S-Figure 7 Applying PCA quality control ellipse and correlation coefficient on hypothetical replicated observations.**


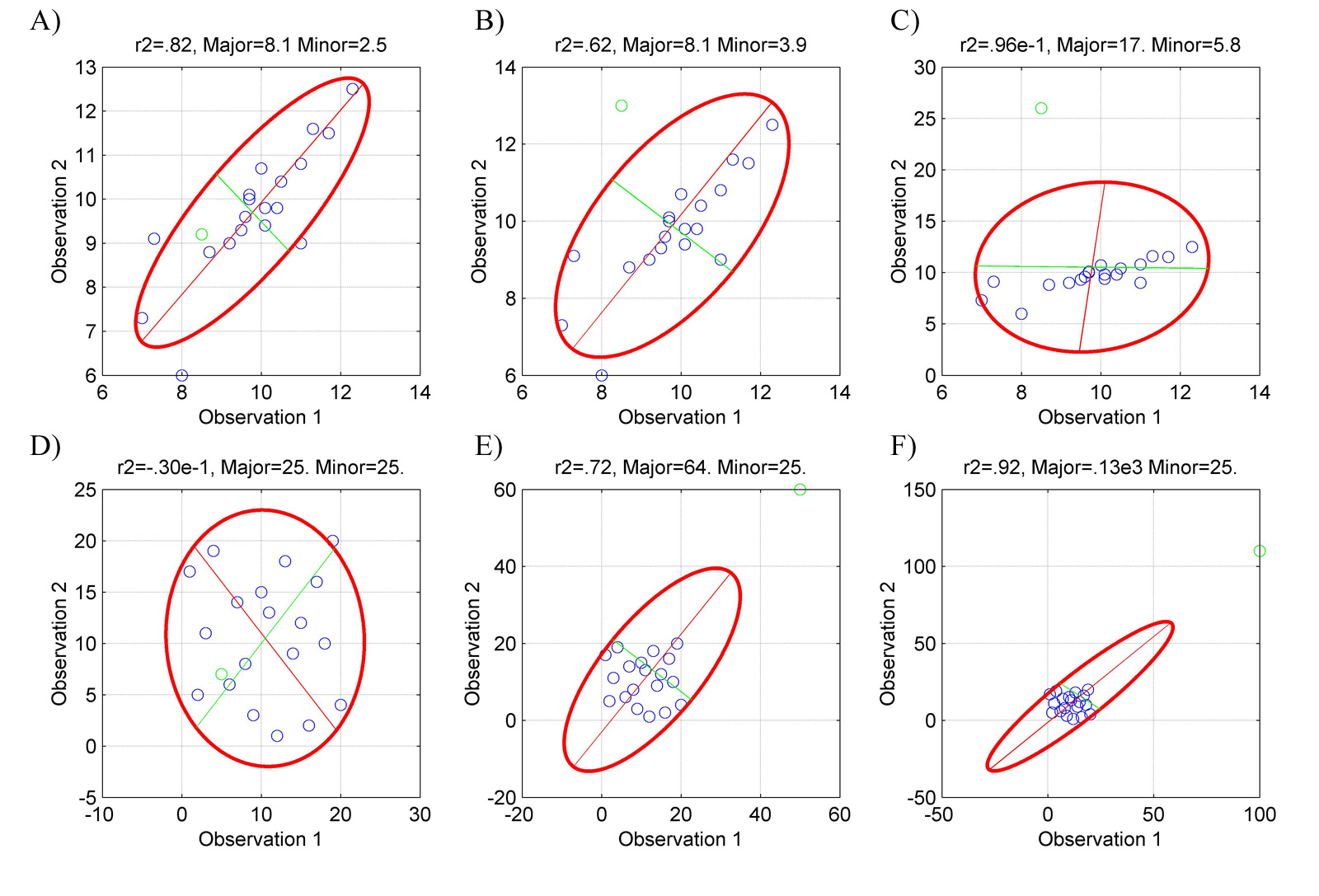


SFigures 7A, 7B, and 7C represent a good agreement between two observations with a green colored data point does not follow the general trend of the rest of the data. SFigures 7D, 7E, and 7F represent a poor agreement between two observations with a green colored data point does not follow the general trend of the rest of the data. In the figure, red ellipse is the PCA quality control ellipse, red smooth line is the major axis of the PCA ellipse, green smooth line is the minor axis of the PCA ellipse, r2 is the correlation coefficient, Major is the length of major axis, and Minor is the length of the minor axis. Here, for well matched two observations, the lower the correlation coefficient and the longer the length of major axis (SFigures 7A, 7B, and 7C); for poorly matched two observations, the higher the correlation coefficient and the longer the length of the major axis (SFigures 7D, 7E, and 7F).

**Supplementary Tables**

**STable1.** **Prediction results of poor PBM quality by using BayesPI2 energy-independent model and energy-dependent model includes dinucleotide interactions.**

|  | TF family | Rank | CorrCoef (Ind) | Length (Ind) | Number (Ind) | CorrCoef (Dep) | Length (Dep) | Number (Dep) |
| --- | --- | --- | --- | --- | --- | --- | --- | --- |
| TF_2 | bZIP | 10 | 0.6 | 12 | 1 | 0.61 | 12 | 1 |
| **TF_21*** | **bHLH** | 15 | **0.55** | 10 | 1 | **0.62** | 12 | 1 |
| TF_41 | NR | 20 | 0.51 | 11 | 1 | 0.54 | 13 | 1 |
| TF_6 | C2H2 ZF (3) | 26 | 0.35 | 11 | 1 | 0.39 | 8 | 1 |
| **TF_10*** | **bZIP** | 28 | **0.5** | 8 | 1 | **0.57** | 9 | 1 |
| TF_29 | C2H2 ZF (9) | 32 | 0.45 | 12 | 1 | 0.45 | 13 | 1 |
| TF_54 | NR | 36 | 0.71 | 12 | 1 | 0.74 | 13 | 1 |
| TF_50 | NR | 37 | 0.6 | 13 | 1 | 0.61 | 11 | 1 |
| TF_20 | Sox | 38 | 0.43 | 9 | 1 | 0.44 | 8 | 1 |
| TF_25 | T-box | 41 | 0.49 | 12 | 1 | 0.48 | 13 | 1 |
| TF_8 | C2H2 ZF (3) | 42 | 0.65 | 13 | 1 | 0.64 | 12 | 1 |
| TF_59 | C2H2 ZF (5) | 44 | 0.45 | 11 | 1 | 0.47 | 12 | 1 |
| **TF_35*** | **bZIP** | 46 | **0.44** | 9 | 1 | **0.54** | 12 | 1 |
| TF_24 | T-box | 47 | 0.45 | 11 | 1 | 0.46 | 11 | 1 |
| TF_34 | AT box | 48 | 0.52 | 9 | 1 | 0.53 | 8 | 1 |
| TF_4 | C2H2 ZF (3) | 49 | 0.39 | 8 | 1 | 0.38 | 9 | 1 |
| TF_1 | NR | 50 | 0.47 | 8 | 1 | 0.5 | 8 | 1 |
| **TF_62** | **C2H2 ZF (5)** | 51 | **0.35** | 13 | 1 | **0.4** | 13 | 1 |
| TF_9 | C2H2 ZF (1) | 52 | 0.41 | 10 | 2 | 0.42 | 12 | 2 |
| TF_61 | C2H2 ZF (6) | 53 | 0.62 | 8 | 1 | 0.63 | 8 | 1 |
| TF_37 | C2H2 ZF (2) | 54 | 0.27 | 10 | 1 | 0.3 | 12 | 1 |
| TF_65 | C2H2 ZF (3) | 56 | 0.3 | 11 | 2 | 0.31 | 11 | 1 |
| TF_57 | SAND | 57 | 0.6 | 8 | 1 | 0.62 | 9 | 1 |
| TF_33 | C2H2 ZF (14) | 58 | 0.25 | 9 | 1 | 0.28 | 12 | 2 |
| **TF_40*** | **NR** | 59 | **0.29** | 9 | 1 | **0.45** | 12 | 2 |
| TF_36 | bZIP | 60 | 0.45 | 9 | 1 | 0.47 | 8 | 1 |
| TF_58 | T-box | 61 | 0.44 | 9 | 1 | 0.45 | 8 | 1 |
| TF_30 | C2H2 ZF (5) | 62 | 0.26 | 10 | 1 | 0.3 | 10 | 1 |
| TF_46 | bHLH | 63 | 0.56 | 8 | 1 | 0.59 | 8 | 1 |
| **TF_66** | **C2H2 ZF (4)** | 64 | **0.18** | 9 | 1 | **0.25** | 13 | 1 |
| TF_60 | C2H2 ZF (14) | 65 | 0.41 | 9 | 1 | 0.43 | 9 | 1 |
| TF_63 | C2H2 ZF (10) | 66 | 0.3 | 8 | 1 | 0.31 | 9 | 1 |

In the table, the 32 TFs were classified by applying fuzzy neuronal gas algorithm on paired PBM quality control parameters (i.e. the length of the major and minor axes of the PCA ellipses), which represents a group of TFs with poor PBM quality; Rank means TFs are sorted in decreasing order of their final performance score across all tested algorithms in Figure 2 of original publication [12]; CorrCoef , Length and Number are Pearson correlation between predicted intensities and testing probe intensities, the length of motif, the first or second motif, respectively; (Ind) and (Dep) represent BayesPI2 energy-independent model and energy-dependent model includes dinucleotide interaction, respectively; TFs marked by star and bold text indicate the increasing of Pearson correlation coefficient is greater than 0.05 by using BayesPI2 energy-dependent model includes dinucleotide interaction energies.

**STable2. Comparison of algorithm performance between good PBM quality and bad PBM quality – Pearson correlation of probe intensities from the DREAM5 challenge**

| Algorithm | P-value | T-value |
| --- | --- | --- |
| Team_D | 1.19E-13 | 9.3883 |
| Team_H | 2.59E-13 | 9.1942 |
| 8mer_pos | 1.58E-12 | 8.7446 |
| 8mer_sum | 2.46E-12 | 8.635 |
| Team_G | 4.75E-12 | 8.5015 |
| 8mer_max | 8.71E-12 | 8.3227 |
| MatrixREDUCE | 9.58E-12 | 8.3268 |
| FeatureREDUCE_dinuc | 1.30E-09 | 7.0894 |
| FeatureREDUCE | 1.72E-09 | 7.0199 |
| Team_F | 3.86E-09 | 6.8191 |
| FeatureREDUCE_PWM | 1.16E-08 | 6.5452 |
| RankMotif++ | 2.06E-08 | 6.4022 |
| FeatureREDUCE_PWM_sec | 2.98E-08 | 6.3087 |
| BEEML-PBM_dinuc | 4.58E-08 | 6.2011 |
| BEEML-PBM_sec | 5.60E-08 | 6.1502 |
| Team_C | 1.13E-07 | 5.9729 |
| Team_J | 4.60E-07 | 5.6126 |
| BEEML-PBM | 5.29E-07 | 5.5765 |
| PWM_align | 3.28E-05 | 4.4687 |
| Team_A | 3.57E-05 | 4.4451 |
| PWM_align_E | 9.72E-05 | 4.1585 |
| Team_E | 0.001209 | 3.3879 |
| Seed-and-Wobble | 0.023779 | 2.3159 |
| Team_I | 0.43028 | -0.79374 |
| Team_B | 0.45019 | 0.75975 |
| Team_K | 0.83471 | 0.20952 |

There are 34 and 32 TFs with good PBM replicates and bad PBM replicates, respectively. Classification of the two groups was based on reproducibility of a pair of training and testing PBM experiments for 66 mouse TFs. Algorithm performance scores (i.e. Pearson correlation of probe intensities) of 26 evaluated methods were adopted from the original publication [1]. In the table, P-value and T-value were obtained by a two tailed T-test for algorithm performance scores between the two groups (i.e. good PBM replicates vs. bad PBM replicates).

**STable3. Comparison of algorithm performance between good PBM quality and bad PBM quality – Pearson correlation of 8-mer intensities from the DREAM5 challenge**

| Name | P-value | T-value |
| --- | --- | --- |
| Team_D | 1.50E-12 | 8.7572 |
| BEEML-PBM_dinuc | 4.30E-12 | 8.4973 |
| 8mer_max | 4.39E-12 | 8.4916 |
| FeatureREDUCE_dinuc | 2.51E-10 | 7.4946 |
| FeatureREDUCE_PWM | 8.04E-10 | 7.2073 |
| Team_G | 1.03E-08 | 6.5756 |
| FeatureREDUCE_PWM_sec | 1.52E-08 | 6.4774 |
| 8mer_pos | 4.11E-08 | 6.2281 |
| BEEML-PBM | 4.97E-08 | 6.1803 |
| BEEML-PBM_sec | 5.18E-08 | 6.17 |
| 8mer_sum | 5.50E-08 | 6.1547 |
| FeatureREDUCE | 8.12E-08 | 6.0561 |
| Team_F | 4.80E-07 | 5.602 |
| Team_H | 8.87E-07 | 5.4424 |
| Team_C | 8.98E-07 | 5.4392 |
| MatrixREDUCE | 1.30E-05 | 4.7251 |
| Team_J | 2.14E-05 | 4.5881 |
| Team_E | 5.30E-05 | 4.333 |
| RankMotif++ | 5.73E-05 | 4.3108 |
| PWM_align_E | 5.94E-05 | 4.3002 |
| PWM_align | 0.000165 | 4.0029 |
| Team_A | 0.001405 | 3.3391 |
| Seed-and-Wobble | 0.005977 | 2.8439 |
| Team_B | 0.032361 | 2.1876 |
| Team_I | 0.72099 | 0.35871 |
| Team_K | 0.72242 | -0.3568 |

There are 34 and 32 TFs with good PBM replicates and bad PBM replicates, respectively. Classification of the two groups was based on reproducibility of a pair of training and testing PBM experiments for 66 mouse TFs. Algorithm performance scores (i.e. Pearson correlation of 8-mer intensities) of 26 evaluated methods were adopted from the original publication [1]. In the table, P-value and T-value were obtained by a two tailed T-test for algorithm performance scores between the two groups (i.e. good PBM replicates vs. bad PBM replicates).

**STable 4. Sorted median Pearson correlation coefficients for BayesPI2 and 26 published algorithms from DREAM5 challenges**

|  | Rank_order | Median_CorrCoef |
| --- | --- | --- |
| Team_E | 1 | 0.72971 |
| Team_D | 2 | 0.69496 |
| FeatureREDUCE | 3 | 0.69435 |
| BEEML-PBM_dinuc | 4 | 0.66035 |
| Team_G | 5 | 0.6531 |
| BEEML-PBM | 6 | 0.6472 |
| FeatureREDUCE_dinuc | 7 | 0.63961 |
| Team_J | 8 | 0.62768 |
| 8mer_pos | 9 | 0.62482 |
| 8mer_sum | 10 | 0.62338 |
| BayesPI2_dinuc | 11 | 0.619 |
| Team_F | 12 | 0.61025 |
| FeatureREDUCE_PWM | 13 | 0.60861 |
| BayesPI2 | 14 | 0.586 |
| Team_I | 15 | 0.58479 |
| BEEML-PBM_sec | 16 | 0.56566 |
| PWM_align_E | 17 | 0.56565 |
| Team_A | 18 | 0.55347 |
| FeatureREDUCE_PWM_sec | 19 | 0.55243 |
| 8mer_max | 20 | 0.54953 |
| MatrixREDUCE | 21 | 0.52944 |
| Team_C | 22 | 0.51621 |
| PWM_align | 23 | 0.51612 |
| Team_H | 24 | 0.48254 |
| Team_K | 25 | 0.4547 |
| Seed-and-Wobble | 26 | 0.31963 |
| RankMotif++ | 27 | 0.26769 |
| Team_B | 28 | 0.26415 |

Pearson correlation coefficients between the predicted probe intensities and the actual intensities for 66 mouse TFs were obtained from the current study (Table 1 and STable 1) and the supplementary Table 3 of original publication [1], respectively. For each algorithm, the median of 66 Pearson correlation coefficients is shown in the Table, where the algorithms were sorted by the median correlation coefficients in descending order. BayesPI2 and BayesPI2_dinuc represent BayesPI2 energy-independent model and energy-dependent model, respectively. Detailed description of other 26 algorithm please refers to the original publication.

**Supplementary Data and Code**

Supporting data sets are available on the website <http://folk.uio.no/junbaiw/CBayesPI2/>, which include quality control parameters of both 66 training PBM and 66 testing PBM experiments, predicted 66 mouse TFs’ binding energy matrices by both BayesPI2 energy-independent and BayesPI2 energy-dependent models, as well as BayesPI2 program and MATLAB code to PCA quality control ellipse.

1. Weirauch MT, Cote A, Norel R, Annala M, Zhao Y, et al. Evaluation of methods for modeling transcription factor sequence specificity. Nat Biotechnol 31: 126-134.
